# Transcriptional induction by ecdysone in *Drosophila* salivary glands involves an increase in chromatin accessibility and acetylation

**DOI:** 10.1093/nar/gkaf284

**Published:** 2025-04-15

**Authors:** Aleksandra A Evdokimova, Tatyana D Kolesnikova, Marina Yu Mazina, Aleksey N Krasnov, Maksim Erokhin, Darya Chetverina, Nadezhda E Vorobyeva

**Affiliations:** Institute of Gene Biology, Russian Academy of Sciences, 119334, Moscow, Russia; Institute of Molecular and Cellular Biology, Siberian Branch of Russian Academy of Sciences, 630090, Novosibirsk, Russia; Institute of Gene Biology, Russian Academy of Sciences, 119334, Moscow, Russia; Institute of Gene Biology, Russian Academy of Sciences, 119334, Moscow, Russia; Institute of Gene Biology, Russian Academy of Sciences, 119334, Moscow, Russia; Institute of Gene Biology, Russian Academy of Sciences, 119334, Moscow, Russia; Institute of Gene Biology, Russian Academy of Sciences, 119334, Moscow, Russia

## Abstract

Transcriptional activation by 20-hydroxyecdysone (20E) in *Drosophila* provides an excellent model for studying tissue-specific responses to steroids. An increase in the 20E concentration regulates the degradation of larval and the proliferation of adult tissues during metamorphosis. To study 20E-dependent transcription, we used the natural system for controlling the 20E concentration—the E23 membrane transporter—which exports 20E from the cell. We artificially expressed E23 in tissues to suppress the first wave of 20E-inducible transcription at metamorphosis. E23 expression revealed a plethora of 20E-dependent genes in salivary glands, while mildly affecting transcription in brain. We described the mechanisms controlling transcriptional activation by 20E in salivary glands. 20E depletion decreased the binding of Pol II and the TFIID subunit, TBP, to the promoters of primary targets, demonstrating the role of 20E in transcription initiation. At target loci, 20E depletion resulted in the malfunctioning of sites co-bound with EcR and CBP/Nejire and enriched for the H3K27Ac mark inherent to active enhancers. At these sites, the 20E concentration was found to control chromatin accessibility and acetylation. We suggest that the activity of these ‘active’ ecdysone-sensitive elements was responsible for the active status of 20E targets in the salivary glands of wandering larvae.

## Graphical Abstract

**Graphical Abstract:**
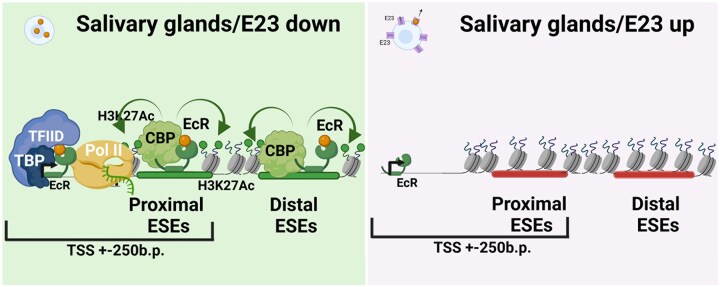

## Introduction

The ability of steroid hormones to directly affect transcription is widely used to treat various pathologies. The main limitation of steroid treatment is the side effects caused by the hormones acting on nontarget tissues. This problem may be resolved by treatment with tissue- or gene-specific regulators of steroid hormones (SERM or SARM, selective modulators of estrogen or androgen receptors activity) [1, 2]. However, the development of these innovative drugs is challenged by the lack of knowledge regarding the tissue-specific action of steroids. It is still not fully understood how different sets of regulatory regions are selected for activation by steroids in various tissues.

Our group considers *Drosophila* to be a promising experimental tool for investigating tissue-specific effects caused by steroids. *Drosophila* has both a simpler steroid-response system (compared with mammals) and well-described tissue-specific phenotypes of steroid action. The main steroid hormone in *Drosophila* is ecdysone, with its active form 20-hydroxyecdysone (20E). An increase in its concentration drives many key stages of fly development. The role of 20E in metamorphosis is best characterized one. Dual 20E peaks during metamorphosis regulate the transition of larva to prepupa and then to pupa, activating fundamentally different transcriptional programs in fly tissues [3–5]. Larval tissues are targeted for destruction by apoptosis or autophagy, while adult tissues begin to proliferate from the imaginal discs, progressing to differentiation [6, 7]. Impressively, all these programs are triggered by 20E.

The *Drosophila* genome contains only one nuclear receptor capable of binding steroids—an ecdysone receptor (EcR) [8, 9]. EcR binds enhancers by partnering with the nuclear receptor Usp and stimulates the activity of these regulatory regions upon 20E binding [10, 11]. It is still not entirely clear whether EcR binding to enhancers is constant or occurs dynamically, exhibiting different binding patterns in different tissues [12, 13]. About ten years ago, convincing experimental evidence emerged concerning the action of 20E in various tissues through the activation of different sets of enhancers [12]. It was proposed that the activation of tissue-specific enhancers by 20E depends on the cooperation between EcR and partner tissue-specific DNA-binding transcription factors. However, no convincing examples of such partnerships have been described to date. There are scant comparative studies of 20E-inducible enhancers in different tissues of living flies. The only exception is a recent study from Professor McKay’s laboratory, which described the binding pattern of EcR in various larval tissues using the Cut&Run technique [14]. They showed a clear difference in EcR genome binding patterns between the salivary glands (larval tissue destined for destruction) and wing imaginal discs (adult tissue destined for further development). The observed correlation between EcR binding sites and increased chromatin accessibility suggested that these EcR binding sites may indeed be tissue-specific enhancers. Thus, the mechanism of tissue-specific enhancer activation by 20E is gradually becoming clearer; whereas the question of how these activated tissue-specific enhancers act on promoters to drive transcriptional activation remains largely unexplored.

Our team previously studied the mechanisms of 20E-dependent transcriptional activation and the transcriptional regulators involved, primarily through studies using cultured cells [11, 15–17]. Our experiments found that the promoters of some well-known 20E target genes are in a high state of readiness for transcription before 20E treatment, a state called RNA polymerase II (Pol II) pause. We demonstrated that factors known to control this ‘pause’, the DSIF and NELF complexes, are involved in maintaining the inactive state of 20E targets as well as being important for genes to achieve their full level of activation after 20E treatment [17]. We suggest that the ‘temporal’ pausing of Pol II during the active transcriptional state contributes to the formation of a high-quality Pol II complex capable of long-term elongation.

In this work, we described the mechanism of 20E-dependent transcriptional activation by studying *Drosophila* tissues. To control the level of 20E in tissues, we used a natural element of the ecdysone cascade—the E23 transporter—which can export 20E from the cells. A negative effect of E23 expression on some known 20E targets in larval tissues was demonstrated previously [18]. E23 expression has also been used as a research tool to describe the role of 20E in the regulation of circadian rhythms [19]. E23 overexpression in pacemaker neurons of the adult fly brain led to the desynchronization of circadian rhythms. However, this method has never been applied to search for 20E targets using genome-wide methods. In the current study, we showed how the expression of this transporter impaired the 20E-dependent transcriptional response. Using this approach, we described the changes occurring at the promoters and enhancers of 20E target genes when the hormone concentration was reduced during the expression of the E23 transporter. That is, we described which working stages of these regulatory regions are under the control of the 20E hormone.

## Materials and methods

### Generation of transgenic *Drosophila* with a cassette for E23 expression

The coding region of the *Drosophila melanogaster e23* gene (which is the same for all the transcripts) was cloned into the construct *attBdir-white-rev-pSK* described previously and marked with a triple FLAG epitope [20]. To control transgene expression, we used a combination of *10xuas* with the *hsp70* minimal promoter [21]. The created cassette was integrated into the attP2 landing site using the phiC31 system [22]. The obtained flies were crossed with *hsp-GAL4 7–1* stock capable of expressing GAL4 under heat shock conditions without leakage at a normal temperature [23]. For brevity, we refer to the resulting stock of *hsp-GAL47-1,CyO/IF;10uas-3xFLAG-E23,w^+^* as *hsp-e23*.

The details of work with dissected larval tissues/cell culture and a description of bioinformatic analysis are provided in Supplementary methods.

### Antibodies

Rabbit polyclonal antibodies against Rpb3 (1–275 aa), CBP/p300/Nejire (125–310 aa); PAF1 (1–234 aa), TBP (1–55 aa) were obtained and described previously in our lab [16, 17, 21]. EcR-C end antibodies against an antigen corresponding to amino acids Ile-691 to Ala-878 of the EcR-B isoform (NM 165465) were described and characterized previously [24]. EcR-full antibodies against full-length EcR-A isoform NM_165 461 were obtained in our lab and described in this manuscript for the first time. To characterize EcR-full antibodies we performed IP procedure with EcR full abs in control and conditions with decreased level of EcR by RNA interference in Drosophila S2 cells (Supplementary Fig. S1A). This experiment demonstrated that EcR full antibodies specifically precipitated protein of the expected size, the amount of which was decreased upon EcR RNAi. Additionally, we characterized previously obtained TBP (1–55 aa) antibodies by RNA interference in *Drosophila* S2 cells (Supplementary Fig. S1B) [21, 25].

All antibodies were affinity-purified. All of them were tested in ChIP experiments using *hsp70* gene induced by heat shock [26]. Antibodies were produced according to the procedures outlined in the NIH (USA) Guide for the Care and Use of Laboratory Animals. The protocol used was approved by the Committee on Bioethics of the Institute of Gene Biology of the Russian Academy of Sciences. All procedures were performed under conditions designed to minimize suffering. Antibodies against histone H3K27Ac (39133) were purchased in Active motif. Antibodies against FLAG epitope were purchased from Sigma-Aldrich (F1804). Anti-lamin (ADL67.10) and anti-actin (JLA20) antibodies were obtained from the Developmental Studies Hybridoma Bank (DSHB). ADL67.10 was deposited to the DSHB by P.A. Fisher.

## Results

### The set of primary 20E target genes includes a membrane transporter E23 capable of exporting 20E from the cell

**Figure 1.**
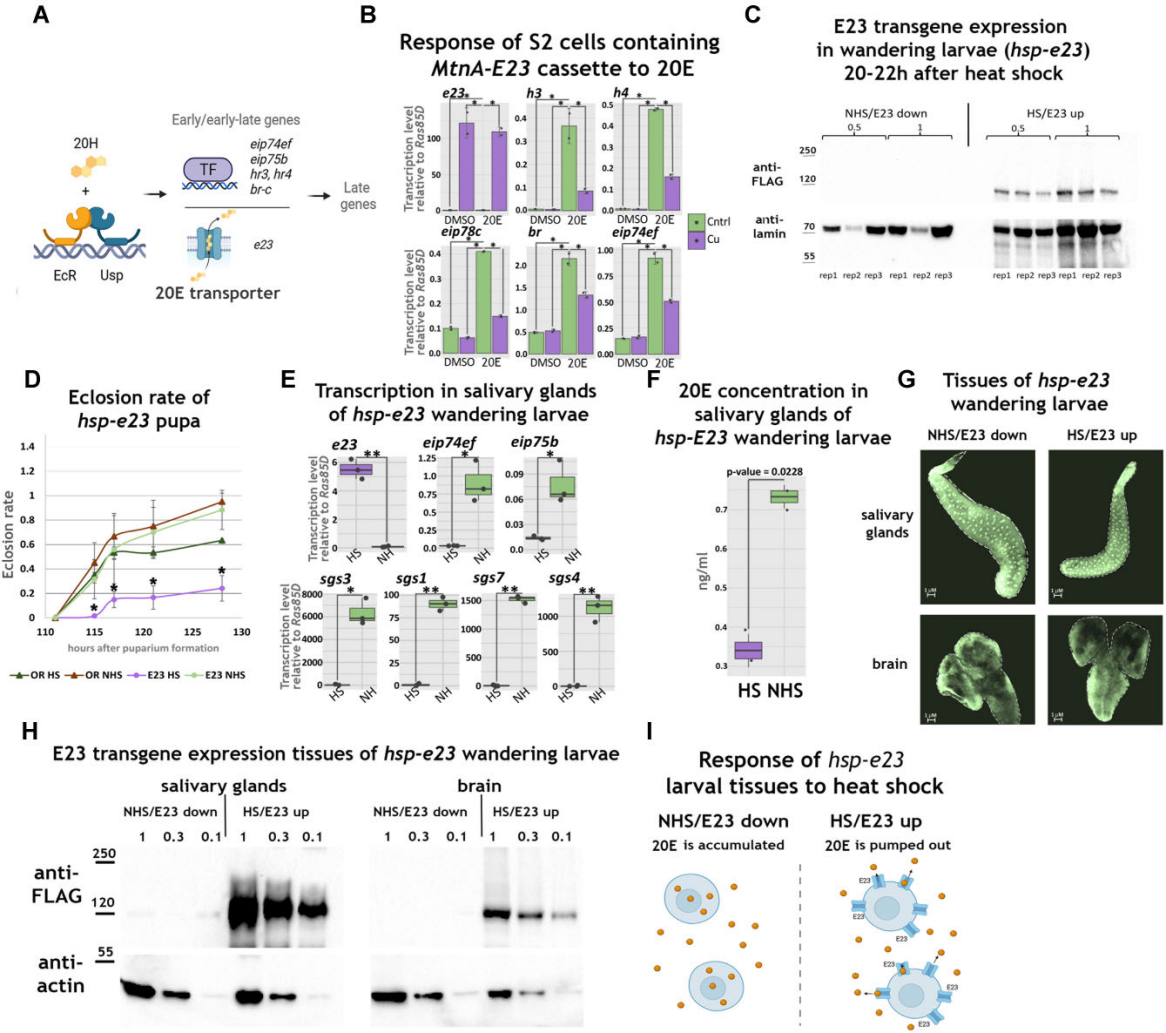
E23 expression in flies results in a phenotype indicating impaired 20E signalling. (**A**) According to the Ashburner model, the primary effect of 20E is the activation of certain transcriptional regulators, which later, during the second 20E peak, lead to the activation of a larger number of target genes. The primary targets of 20E include the membrane transporter E23. This scheme and following were created using *BioRender*(https://biorender.com/rvaehc4). (**B**) Transcriptional level of described 20E targets were assessed in S2 *Drosophila* cells bearing a cassette for the expression of E23 upon CuSO_4_ treatment (purple marks data from CuSO_4_-treated cell, and green, untreated). Transcriptional level was measured by quantitative real-time PCR (qRT-PCR) both in cells treated with 0.3 μM 20E for 1-h and in control cells (dimethyl sulfoxide/DMSO-treated) and presented as a level relative to *ras85D* transcription. (**C**) Western blot analysis of total protein extracts derived from the full *hsp-e23* wandering larvae treated with double 1-h heat shock 20–22 h before pupariation (with 1-h rest between) (HS) or from untreated ones (NHS). Extract was loaded with dilutions (0.5 and 1) and in triple replicates. Western blots were stained with anti-FLAG (to detect FLAG-tagged E23) or anti-lamin antibodies. (**D**) Eclosion rate of *hsp-e23* flies was determined after performing a 1-h treatment with the heat shock of white prepupae (E23 HS) and without the treatment (E23 NHS). Eclosion time is presented starting from the puparium formation. Eclosion rate is presented as a portion of eclosed flies relative to a total number taken for the experiment. Control experiment with the same scheme was made for the *Oregon* flies (OR HS, and OR NHS). (**E**) Transcriptional levels of described 20E targets were assessed in salivary glands of *hsp-e23* wandering larvae (collected 4–6h before pupariation) after their treatment with double 1-h heat shock 20–22 h before pupariation (with 1-h rest between) (HS) or untreated ones (NHS). Transcriptional level was measured by qRT-PCR and presented as a level relative to *ras85D* transcription. (**F**) 20E concentration was assessed by liquid chromatography-mass spectrometry (LC-MS) in methanol extract of salivary glands dissected from *hsp-e23* wandering larvae after their treatment with double 1-h heat shock 20–22 h before pupariation (with 1-h rest between) (HS) or from untreated ones (NHS). Data values are presented as points. Experiment was performed in two biological replicas and a *t*-test was performed to compare the means of the two groups (NHS and HS). The *P*-value (0.0228) is provided. (**G**) Salivary glands and brain dissected from *hsp-e23* wandering larvae (collected at 4–6 h before pupariation) after their treatment with double 1-h heat shock 20–22 h before pupariation (with 1-h rest between) (HS) or from untreated ones (NHS). Tissues were fixed with formaldehyde and stained with 4′,6-diamidino-2-phenylindole/DAPI. (**H**) Western blot analysis of total protein extract derived from salivary glands or brain of *hsp-e23* wandering larvae treated with double 1-h heat shock 6 h before the dissection (with 1-h rest between) (HS) or from untreated ones (NHS). Extract was loaded as series of dilution (1, 0.3, and 0.1). Western blots were stained with anti-FLAG (to detect FLAG-tagged E23) or anti-actin antibodies. (**I**) Schematic depicting the effect of artificial E23 expression (achieved by a heat shock treatment of *hsp-e23* larvae) on intracellular 20E levels in *Drosophila* tissues. Created in BioRender (https://BioRender.com/po0vuvo). In panels (B), (D), and (E) the error bars represent the standard deviation. The experiments were performed as triplicate [duplicate in panel (B)]. Individual measurements presented as points. Statistical significance analysis was performed using the paired *t*-test (* means *P* ≤ .05, and ** means *P* ≤ .01).

According to the Ashburner model, the effect of 20E on the cells of wandering larvae is implemented through a gene cascade, called the ecdysone cascade [27, 28]. The primary effect of 20E is the activation of certain transcriptional regulators, which later, during the second 20E peak, lead to the activation of a larger number of target genes. The primary targets of 20E included not only DNA-binding transcription factors but also the membrane transporter E23, which turned out to be an ecdysone transporter (Fig. 1A) [29]. The artificial overexpression of the E23 transporter during the 20E response interferes with 20E-dependent transcriptional activation [18]. Moreover, E23 expression prevented 20E-dependent transcription of an artificial reporter consisting of EcR-bound sites in a dose-dependent manner [19]. The expression of this protein as part of the natural ecdysone cascade promotes a rapid decrease in the 20E concentration in cells, which is a condition for the induction of early-late transcription factors [25, 30].

To additionally characterize the ability of E23 to prevent 20E-inducible transcription, we created a stable S2 cell line expressing E23 under the control of the *MtnA* promoter. We compared this cell line’s ability to respond to 1-h 20E treatment under both elevated E23 expression—stimulated by the presence of CuSO_4_—and control conditions (Fig. 1B). The transcriptional response to 20E was assessed by measuring the transcriptional levels of genes known to be rapidly induced in S2 cells upon 20E treatment [31]. For these 20E-activated genes, E23 expression significantly reduced their degree of 20E activation, thus demonstrating the ability of E23 to prevent 20E-dependent transcriptional induction in cultured cells.

Importantly, when E23 was expressed in cultured S2 cells, the protein accumulated outside the nucleus (Supplementary Fig. S2). Immunostaining results support that E23 is not a nuclear protein and, therefore, its effect on the 20E response is not mediated by its ability to bind DNA and act as a transcriptional regulator.

### E23 expression in flies results in a phenotype indicating impaired 20E signalling

We used artificial E23 expression as an experimental tool to study the effect of 20E depletion in *Drosophila* tissues. To this end, we produced transgenic flies by integrating a cassette containing the coding region of *e23* fused with the 3xFLAG epitope under the control of 10xUAS sites (integrated into the attP2 landing site using the phiC31 system) [22].

To study the phenotype arising from the artificial expression of E23, we crossed flies bearing the E23 expression construct (*10UAS-3xFLAG-E23,w+*—hereon referred to as *uas-e23*) with two alternative drivers expressing tissue-specific GAL4 (Supplementary Fig. S3). Thus, we crossed *uas-e23* flies with the *fkh*-GAL4 driver, which expresses GAL4 in the salivary glands starting at an embryonic development stage. This resulted in a significant decrease in the size of the salivary glands at the wandering larval stage, demonstrating the involvement of ecdysone signalling in their developmental control. This effect of E23 corresponds closely with the phenotype caused by EcR RNA interference in the salivary glands [14]. Crossing of *uas-e23* flies with the *tj*-GAL4 driver (stimulating expression in somatic follicular cells of the ovary) led to the arrest of *Drosophila* oogenesis at stage 8. This is also consistent with the phenotype observed in flies with a defect in ecdysone signalling [32, 33]. Upon E23 expression, we found no follicles with migrated follicular cells (20E was previously shown to control migration) [34].

Next, to specifically control the E23 expression stage, the *uas-e23* flies were crossed with the *hsp-GAL4 7–1* stock capable of expressing GAL4 under heat-shock conditions without leakage at normal temperatures [23]. For brevity, we refer to the resulting stock of *hsp-GAL4 7–1,CyO/IF;10UAS-3xFLAG-E23,w^+^* as *hsp-e23*.

E23 protein expression in the resulting *hsp-e23* flies persisted for at least 22 h after larvae were treated with double 1-h heat shocks with a 1-h rest interval (Fig. 1C). We detected no E23 FLAG-tagged protein in nonheat-shocked L3 wandering larvae. Double heat-shock treatment of L3 larvae 20–22 h before puparium formation did not affect the ratio of wandering larvae. This result aligns with the general view on the control of wandering behaviour, which is partially regulated by 20E, but is initiated earlier than 22 h before puparium formation. Yet, 1-h heat-shock treatment of *hsp-e23* white prepupae produced a critical effect on fly development (Fig. 1D). The number of eclosed *hsp-e23* flies was dramatically reduced after heat-shock treatment; heat shock had a much smaller effect on wild-type *Oregon* flies.

Next, we characterized the effect of the E23 transporter on the transcription of 20E target genes using the salivary glands of *hsp-e23* flies, the tissue where the ecdysone cascade is best described. We treated *hsp-e23* larvae with double heat shocks 20–22 h before puparium formation. Salivary glands were collected for analysis 4–6 h before puparium formation, during the first major 20E metamorphosis peak. Following heat shocks, there was a substantial decrease in the transcription of the selected 20E target genes involved in the ecdysone cascade and salivary gland secretion (Fig. 1E).

To assess the direct influence of E23 expression on 20E tissue concentrations, the hormone was methanol-extracted from the salivary glands of heat-shock treated and untreated larvae (according to the scheme described for the experiment in Fig. 1E); concentrations were estimated using LC-MS with 20E (H5142 Sigma) as the reference (Fig. 1F). A decrease in 20E levels in the salivary glands after double heat shock was detected. Considering that E23 does not destroy 20E but rather exports it, we believe that the effect of E23 on intracellular concentrations may be much greater.

To estimate the tissue-specific response to 20E, two different tissues were selected for further experiments: salivary glands and brain. Treatment of *hsp-e23* larvae with double heat shocks 20–22 h before puparium formation did not affect the appearance of these tissues (assessed 4–6 h before pupariation) (Fig. 1G). Only the salivary glands became slightly smaller after heat-shock treatment, which we associated with them containing reduced quantities of secretions. The expression of the FLAG-E23 protein in both target tissues after heat shock was confirmed by western blotting (Fig. 1H).

The successful detection of E23 expression in target tissues of *hsp-e23* larvae, as well as the absence of major developmental defects upon heat-shock treatment, confirms their suitability for studying the tissue-specific effect of 20E depletion caused by E23 expression (Fig. 1I).

### Artificial E23 expression alters the transcription of hundreds of targets in the salivary glands of wandering larvae, having a much weaker effect on transcription in the brain

**Figure 2.**
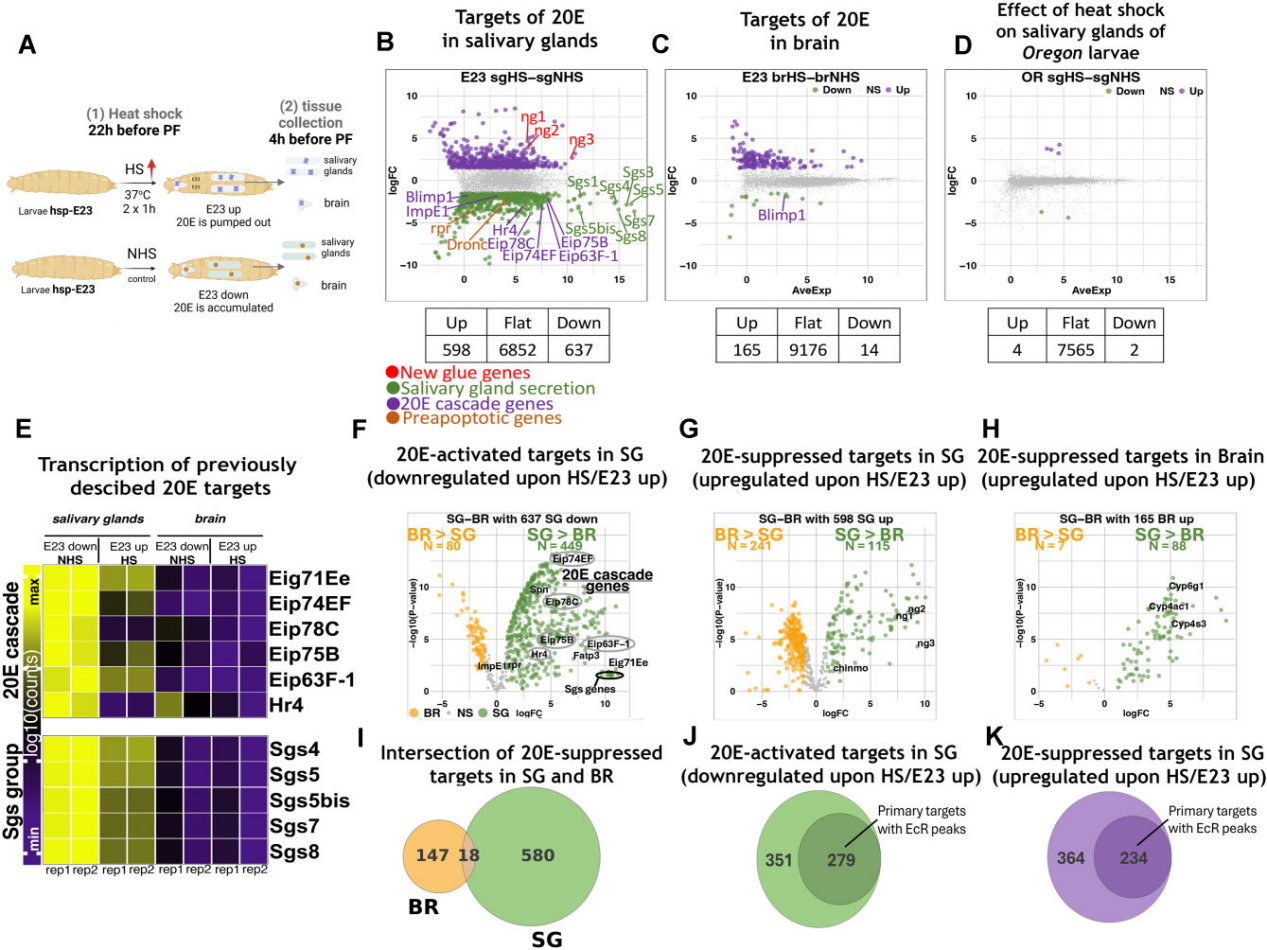
Artificial E23 expression alters the transcription of hundreds of targets in the salivary glands of wandering larvae, having a much weaker effect on transcription in the brain. (**A**) A scheme describing the experimental procedures performed on *hsp-e23* larvae to determine the influence of E23 artificial expression on tissue transcriptional response to 20E at the wandering larval stage. The *hsp-e23* larvae were treated with double 1-h heat shock 20–22 h before pupariation (with 1-h rest between) (HS) to induce E23 expression or untreated (NHS). Tissues were collected at the wandering larval stage ∼4–6 h before pupariation and subjected to RNA-seq analysis. Created in BioRender (https://BioRender.com/u7lvrpk) (**B**) and (**C**) Mean-difference plots (MD-plots) representing the effect of E23 expression on transcriptional levels of genes in salivary glands (**B**) or brain (**C**) of *hsp-e23* wandering larvae in response to their treatment with heat shocks according to the scheme presented in panel (A). Genes whose level was changed upon heat shock treatment and E23 expression (with log fold change (FC) ≥|1.5|; adj. *P* ≤ .05) were recognized as 20E target genes in corresponded tissues. (**D**) MD-plot representing the effect of heat shocks treatment according to the scheme (**A**) on transcriptional levels of genes in salivary glands of *Oregon* wandering larvae. (**E**) Heatmap representing the transcriptional level of previously described 20E target genes in salivary glands and brain of *hsp-e23* wandering larvae treated according to the scheme (**A**). Signal represents the log_10_ number of read counts. Experiments were performed in duplicates. (**F**) Volcano plot representing the comparative analysis of transcription levels of 20E-activated primary targets of salivary glands (downregulated upon E23 expression) in two tissues: salivary glands and brain, assessed in control untreated conditions in *hsp-e23* wandering larvae. (**G**) and (**H**) Volcano plots representing the comparative analysis of transcription of 20E-suppressed targets (upregulated by the E23 expression) of salivary glands (**G**) and brain (**H**) in two tissues (salivary glands and brain of *hsp-e23*), assessed in control untreated conditions. (**I**) Venn diagram reflecting the intersection between pools of salivary glands and brain 20E-suppressed targets (activated upon E23 expression). (**J**) and (**K**) Venn diagrams showing a portion of primary 20E targets (defined as targets bearing EcR peaks in their loci) in a total number of target genes downregulated (**J**) and upregulated (**K**) in salivary glands of *hsp-e23* wandering larvae upon their treatment according to the scheme (**A**). SG, salivary glands, BR, brain, 20E, 20-hydroxyecdysone; HS, heat shock; NHS, nonheat shock.

To estimate the tissue-specific target genes controlled by 20E, we conducted experiments according to the following scheme (Fig. 2A). We aimed to prevent transcriptional induction resulting from an increase in 20E levels during the wandering larval stage by expressing the E23 transporter during the preliminary developmental stages. *hsp-e23* larvae were subjected to double 1-h heat shocks at 37°C 20–22 h before puparium formation with a 1-h rest interval at 25°C; larvae were then allowed to grow under normal conditions. Tissues for analysis were collected 4–6 h before puparium formation (during the wandering larval stage).

RNA-seq analysis was conducted on the salivary glands and brain of both heat-shocked and nonheat-shocked *hsp-e23* larvae. The main purpose of this analysis was to identify target genes whose transcription is dependent on 20E level, i.e. activated upon an increase in 20E concentrations (Fig. 2B and C). Surprisingly, artificial E23 expression resulted in the reduced transcription of 637 target genes (630 after removing the transposons) in the salivary glands, while there were only 14 reduced-transcription targets in the brain (log FC ≥ |1.5|; adj. *P ≤*.05) (Supplementary Tables S1–S3). This result demonstrated the resistance of transcription in the brain to E23 expression and decreased 20E concentrations (or it may be due to lower artificial E23 expression levels in this tissue). The salivary gland genes with reduced transcription were consistent with our expectations. E23 expression reduced the transcription of previously described 20E targets—the ecdysone cascade *(Eip74EF*, *Eip75B*, *Eip78C*, and *Eip63EF-1)*, salivary gland secretion (*Sgs1*, *Sgs3*, *Sgs4*, etc.), and apoptotic genes (*Rpr* and *Dronc*) [14, 27, 35]. Accordingly, Gene Set Enrichment Analysis (GSEA) of 20E-activated targets in the salivary glands (downregulated upon E23 expression) revealed enriched categories: apoptosis (which includes ecdysone cascade genes), dorsal-ventral axis formation, and the Hippo pathway (Supplementary Fig. S4). The latter is closely connected to ecdysone signalling through coregulators Tai and Yki [36–38].

A significant number of 20E-suppressed genes, whose transcription was activated in response to E23 expression, were found both for salivary glands (N = 598) and brain (N = 165) (log FC ≥|1.5|; adj. *P* ≤ .05) (Supplementary Tables S4 and S5). Among the genes upregulated in salivary glands, we observed the new glue gene group (*ng1*, *ng2*, and *ng3*). The GSEA of 20E-suppressed targets in the salivary glands (upregulated upon E23 expression) revealed enriched categories: basal transcriptional factors, pentose-phosphate pathway, and glycolysis, which is consistent with a previously described repressive effect of 20E (Supplementary Fig. S5) [24, 39]. While 20E-suppressed genes in brain fell into the categories of DNA replication, homologous recombination, and glycolysis (Supplementary Fig. S6).

The control RNA-seq analysis of *Oregon* flies revealed that transcriptional changes in salivary gland targets were not associated with the method of E23 expression (heat-shock treatment). RNA-seq analysis of the salivary glands of Oregon (wild-type) larvae similarly heat-shock treated revealed that only six genes had an altered transcription after heat shock (Fig. 2D) (Supplementary Table S6). We did not conduct a similar control experiment in brain due to the low number of altered targets.

When analysing the effect of E23, we observed that the transcription of ecdysone cascade genes was higher in the salivary glands than in brain under normal conditions (without heat shock) (Fig. 2E). In other words, previously described 20E targets were suppressed in wandering larvae brain compared with the salivary glands. The lack of an effect of E23 expression on the transcription of ecdysone cascade genes in brain could be explained by the fact that these genes are simply not transcribed in this tissue at this larval stage.

Additionally, most of the 20E targets revealed in the salivary glands and brain demonstrated a tissue-specific transcription pattern (assessed by RNA-seq data analysis of salivary glands and brain under control, nonheat-shocked conditions). The prevailing number of 20E-activated genes, with reduced transcriptional levels in the salivary glands upon E23 expression, was found to have a much higher transcriptional level in salivary glands compared with brain (Fig. 2F). In contrast, 20E-suppressed targets in salivary glands demonstrated higher transcriptional levels in brain (Fig. 2G). Furthermore, the 20E-suppressed target genes in brain, activated upon E23 expression, were found to be salivary-gland enriched (Fig. 2H and I). The tissue-specific activity of genes regulated by E23 expression suggests that the 20E concentration regulates a ‘tissue-specificity’ barrier. E23 expression in both salivary glands and brain leads to the activation of gene pools that are normally transcribed in other tissues at this particular stage.

Overall, our search for 20E targets in tissues of wandering larvae revealed the mild effect of E23 expression on transcription in the brain. The observed lower transcription levels of well-known ecdysone cascade genes in the brain compared with the salivary glands may indicate a lack of ecdysone activity in this tissue at this particular stage.

Furthermore, we decided to focus on the molecular mechanisms underlying the response to 20E in the salivary glands, which demonstrated the expected response to E23 expression. To this end, we selected the primary 20E-activated targets from among the 630 genes decreased upon E23 expression and 20E depletion in the salivary glands. We considered ‘primary targets’ those genes with 20E-dependent transcription and whose loci (with the flanking regions ±5 kb) have EcR peaks (which can be directly affected by 20E). To estimate genome-wide EcR binding, we performed ChIP-Seq on the salivary glands of *hsp-e23* wandering larvae with antibodies against full-length EcR (obtained in our lab). The binding profile of these antibodies in the genome and the total number of EcR peaks correlated with the ChIP-Seq obtained with other EcR-C end antibodies (also obtained in our lab and described previously; for details, please consult the ‘Materials and methods’ section) [24]. This approach of selecting primary target genes allowed us to sort 279 20E-activated primary targets in the salivary glands of wandering larvae, identified by artificial E23 expression (Fig. 2J) (Supplementary Table S7). Using a similar approach, we also selected 234 20E-suppressed primary targets in salivary glands from genes whose transcription is activated upon E23 expression (Fig. 2K) (Supplementary Table S8).

### E23 expression and 20E depletion leads to decreased Pol II and TBP binding at the transcriptional start sites of 20E-activated primary targets in the salivary glands

To study the mechanism of transcriptional induction by 20E, we characterized the molecular state of promoters upon 20E depletion induced by E23 expression in the salivary glands of *hsp-e23* wandering larvae (according to the scheme in Fig. 2A). From all the promoters (corresponding to 279 20E-activated primary target genes in salivary glands), we selected 164 unique transcriptional start sites (TSSs) exhibiting a reduced RNA-seq signal within 500 bp downstream of TSSs (with FC ≥|1.5|; *P* ≤ .05) (these TSSs were named 20E-activated TSSs and are listed in the Supplementary materials as Supplementary Tables S9 and S10). We attributed the lower number of 20E-activated TSSs compared with the number of loci to the loss of low-expressed targets in the analysis (insufficient number of reads proximal to TSS regions).

**Figure 3.**
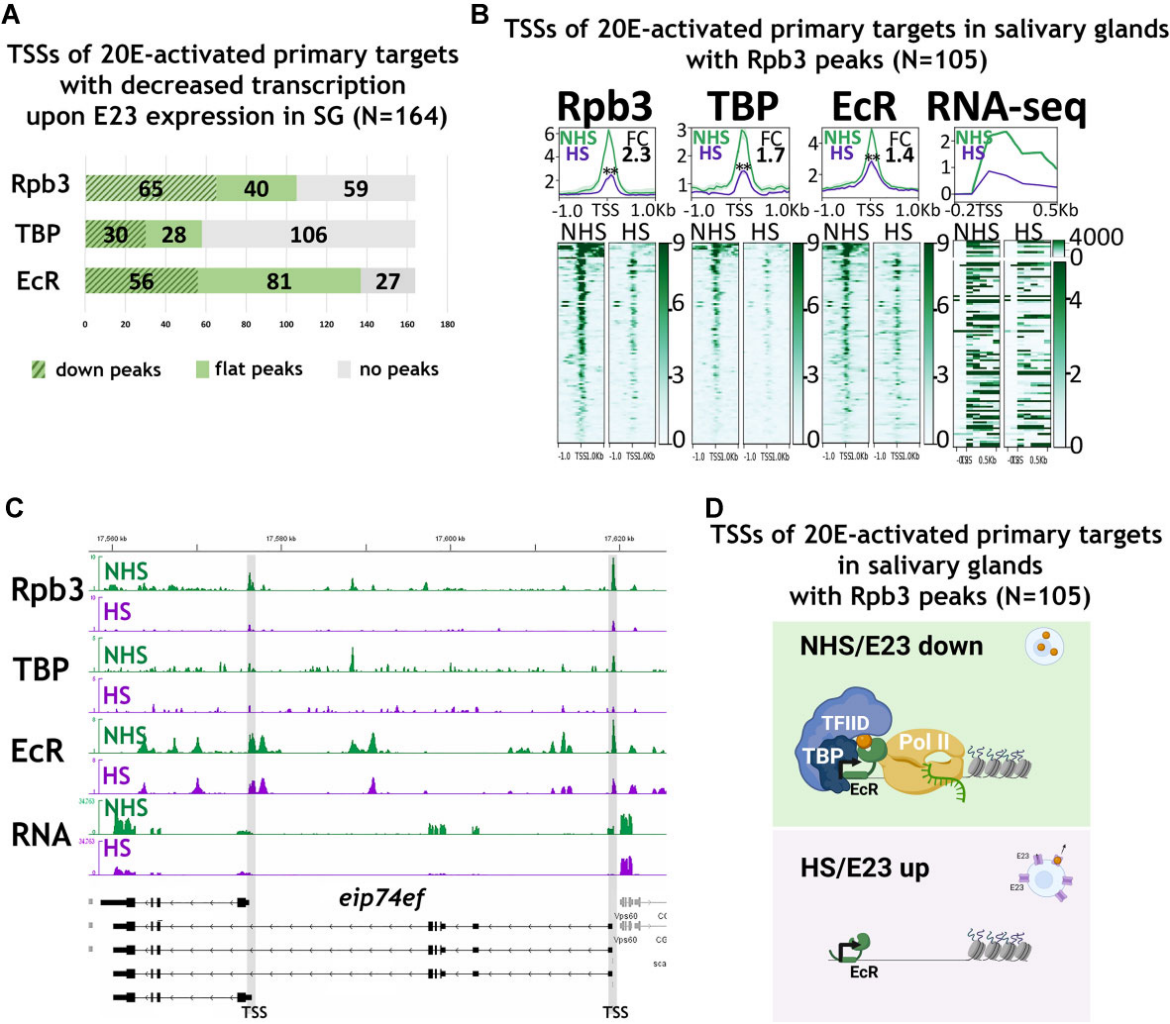
E23 expression and 20E depletion leads to decreased Pol II and TBP binding at the TSSs of 20E-activated primary targets in the salivary glands. (**A**) Chart representing the changes in a distribution of Rpb3, TBP, and EcR peaks at 20E-activated TSSs in salivary glands of *hsp-e23* wandering larvae upon their pre-treatment with heat shock (according to a scheme at Fig. 2A). The chart cells represent the number of TSSs associated with Rpb3, TBP, and EcR peaks, categorized into those with downregulated peaks (striped), unchanged or flat peaks, and no detectable peaks which was estimated by the DiffBind algorithm (FC|≥1|; *P* ≤ .05). (**B**) Average distribution of Rpb3, TBP, EcR proteins estimated by ChIP-Seqs at 20E-activated TSSs in salivary glands, bearing Rpb3 peaks, (N = 105) in control nonheat-shocked condition (NHS) and after treatment of *hsp-e23* larvae 20–22 h before pupariation with double 1-h heat shock (with a 1-h rest at room temperature/RT) (HS). ChIP-Seq binding level was calculated as a ratio to Input. RNA-seq signal is presented as a number of reads normalized to the genome. The X-axis represents the distance to the TSS in kbp. Average profiles were calculated as a median of binding level with the standard error displayed on the profiles. The FC was calculated using normalized coverage within 500 bp around the center of the analysed TSSs (as a ratio of NHS signal to HS signal). The results of the paired *t*-test analysis are provided on the graphs, where ‘**’ means *P* ≤ .01. (**C**) Binding profiles of Rpb3, TBP, and EcR estimated by ChIP-Seqs and an average of RNA-seq signal in a locus of *eip74ef* gene (an example of 20E-activated primary target in SG) in control salivary glands (NHS: nonheat shock) and treated salivary glands (HS: heat-shock) of *hsp-e23* wandering larvae. (**D**) A model illustrating an effect of 20E depletion (achieved by E23 expression) on the promoters of 20E-activated primary targets in salivary glands. E23 expression via heat shock treatment (HS) 20–22 h before pupariation impairs a recruitment of Rpb3 and TBP to TSSs of 20E-activated primary targets suggesting the impact of 20E in the transcription initiation. Created in BioRender (https://BioRender.com/spo2c4b).

To characterize the selected TSSs of 20E-activated genes, we analysed the presence of Rpb3, TBP, and EcR peaks and their changes upon E23 expression and 20E depletion in salivary glands (Fig. 3A and Supplementary Fig. S7). The differential protein binding was assessed using ChIP-Seq and analysed using the DiffBind package and DESeq2 method (with FC|≥1|; *P* ≤ .05) [40]. All ChIP-Seq data were obtained for two biological replicates of each condition (correlation tests are in Supplementary figures). We observed Rpb3 peaks at 105 of the 164 20E-activated TSSs that were downregulated upon E23 expression. More than half of these had a statistically significant reduction in binding upon E23 expression (65 out of 105 Rpb3 peaks at 20E-activated TSSs). TBP bound to 58 of the 164 20E-activated TSSs (with 30 peaks exhibiting decreased binding upon E23 expression). Additionally, EcR was detected at 137 of the 164 20E-activated TSSs in salivary glands, with 56 peaks showing decreased binding under treatment conditions. The absence of Rpb3 and TBP binding at some 20E-activated TSSs could be due to a limitation of the ChIP-Seq method. The sensitivity of our antibodies was likely insufficient to detect peaks at all promoters.

Nonetheless, our data allowed us to assess the influence of E23 expression in salivary glands on 105 20E-activated TSSs bearing Rpb3 peaks under nonheat-shocked conditions. We analysed the molecular changes at the 105 20E-activated TSSs in salivary glands by plotting the average binding in both heat-shocked and control conditions (Fig. 3B) (Supplementary Table S11). We observed a substantial decrease in Rpb3 binding at the analysed TSSs upon E23 expression, demonstrating a contribution of 20E to Rpb3 recruitment at the promoters of 20E-activated targets in salivary glands. A concomitant decrease in TBP binding upon E23 expression indicated that 20E depletion may affect Pol II recruitment by influencing the initiation transcription stage [via TBP as an essential subunit of the preinitiation complex (PIC)]. The presence of EcR at the 20E-activated TSSs in salivary glands suggests its direct involvement in PIC recruitment and even the existence of some undescribed physical contacts.

The changes in the state of promoters caused by E23 expression are clearly visible in the profiles at the *eip74ef* gene (Fig. 3C). We detected a significant reduction of Rpb3 and TBP binding at 20E-activated TSSs. The decrease in EcR binding at TSSs upon 20E depletion was milder.

The observed decrease in Rpb3 and TBP binding at 20E-activated TSSs does not form part of the overall changes in their binding to TSSs throughout the genome. No decrease in average Rpb3, TBP, and EcR binding levels was detected when assessing all *Drosophila* TSSs (Supplementary Fig. S8). In addition, there was no decreased binding of these proteins to 259 20E-suppressed TSSs of genes upregulated upon E23 expression in the salivary glands (selected from the 234 20E-suppressed loci by assessing the RNA-seq using the scheme described for 20E-activated TSSs) (Supplementary Fig. S9) (Supplementary Tables S12 and S13). We did not find a significant increase in Rpb3 and TBP binding to 20E-suppressed TSSs upon E23 expression, demonstrating that the suppressive effect of 20E on promoters is not through influencing the Rpb3 recruitment rate but probably via the control of RNA Pol II promoter escape.

The obtained data allow us to propose a model describing how 20E promotes the transcription of 20E-activated TSSs in the salivary glands (Fig. 3D). 20E depletion via E23 expression impaired Rpb3 recruitment to the activated TSSs; the concomitant loss of TBP binding showed that the influence of 20E on Rpb3 recruitment may be through the transcription initiation stage. The observed binding of EcR to 20E-activated TSSs signifies a possible direct influence of EcR and 20E on the preinitiation machinery.

Based on our assumption that the ability of 20E to activate transcription of one set of genes while suppressing another may be associated with differences in the promoter structure of these gene groups, we examined the enrichment in promoter-associated motifs (Supplementary Fig. S10). This analysis was performed for all 20E-activated (N = 164) and 20E-suppressed (N = 259) TSSs, using recent data on the distribution of motifs in all *Drosophila* promoters (for details, please consult the Supplementary methods section) [41]. Compared to both 20E-suppressed TSSs and the control set representing all of the genome’s TSSs, 20E-activated TSSs were found enriched with TATA-box and GAGA motifs. Since 20E-activated genes are tissue-specific (enriched in salivary glands), the presence of these motifs was expected [42–44]. Our promoter-associated motif analysis yielded results that were similar to those from the TBP ChIP-Seq analysis. Compared to 20E-suppressed TSSs, the average TBP binding level was elevated at 20E-activated TSSs (Supplementary Fig. S10). These results indicate that 20E positively regulates TATA box-rich and TBP-bound promoters, which is to be expected for a key development regulator and has indeed been observed previously in cell culture experiments [45].

In contrast, 20E-suppressed TSSs did not show any enrichment of TATA or GAGA motifs, but of the Pause button motif instead (Supplementary Fig. S10). The presence of the Pause button motif in the cohort of 20E-suppressed TSSs may be associated with the above-described control mechanism of their transcription by 20E via RNA Pol II promoter escape.

### 20E-activated promoters are tissue-specifically transcribed in salivary glands but retain some Pol II peaks in the brain, where they are less active

**Figure 4.**
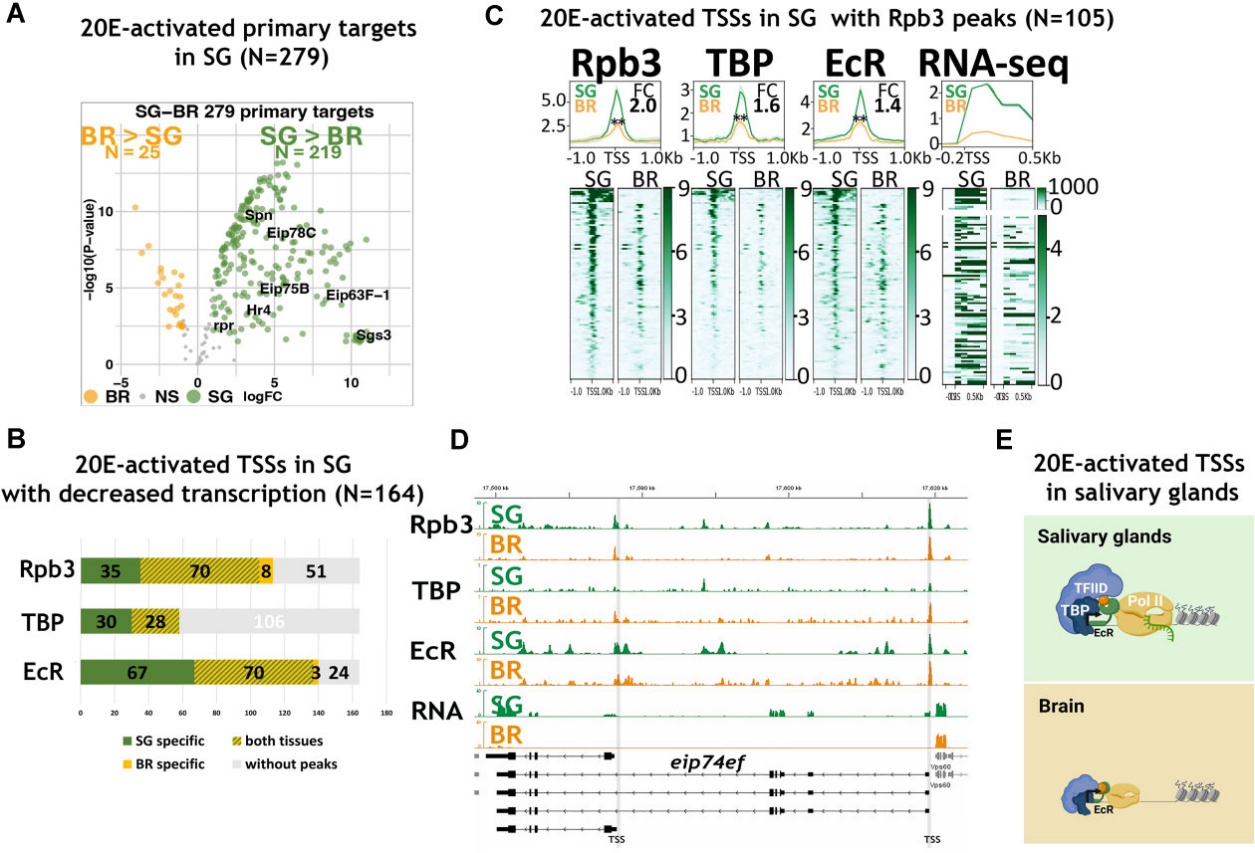
20E-activated promoters are tissue-specifically transcribed in salivary glands but retain some Pol II peaks in the brain, where they are less active. (**A**) Volcano plot representing the comparative analysis of transcription levels of 20E-activated primary targets of salivary glands (downregulated upon E23 expression) in two tissues: salivary glands and brain, assessed in control untreated conditions in *hsp-e23* wandering larvae. (**B**) Chart representing the distribution of Rpb3, TBP, EcR peaks at 20E-activated TSSs of salivary glands in two tissues: salivary glands and brain. The chart presents the number of TSSs associated with Rpb3, TBP, and EcR peaks, categorized into those specific for salivary glands (SG specific), specific for brain (BR specific) and those that are present in both tissues (striped). (**C**) Average distribution of Rpb3, TBP, EcR proteins estimated by ChIP-Seqs at 20E-activated TSSs of salivary glands, bearing Rpb3 peaks, in not-treated salivary glands (SG) and brain (BR) of *hsp-e23* wandering larvae. ChIP-Seq binding level was calculated as a ratio to Input. RNA-seq signal is presented as a number of reads normalized to the genome. The X-axis represents the distance to the TSS in kbp. Average profiles were calculated as a median of binding level with the standard error displayed on the profiles. The FC was calculated using normalized coverage within 500 bp around the center of the analysed TSSs (as a ratio of SG signal to BR signal). The results of the paired *t*-test analysis are provided on the graphs, where ‘**’ means *P* ≤ .01. (**D**) Binding profiles of Rpb3, TBP and EcR estimated by ChIP-Seqs and an average of RNA-seq signal in a locus of *eip74ef* gene (an example of 20E-activated primary target in SG) in salivary glands (SG) and brain (BR) of *hsp-e23* wandering larvae (in untreated conditions). (**E**) Model illustrating a tissue-specificity of a transcriptional response to 20E. Elevated binding level of Rpb3, TBP, and EcR at 20E-activated TSSs in salivary glands suggests that increased transcriptional levels in salivary glands are due to enhanced Rpb3 recruitment and RNA Pol II promoter escape. Created in BioRender (https://BioRender.com/5llvn5z).

In Fig. 2, we report the tissue-specific transcription pattern of 20E-activated targets in the salivary glands. Expectedly, 20E-activated primary target genes (bearing EcR peaks within their loci ±5 kb), also exhibited tissue-specific salivary gland-enriched transcriptional patterns (Fig. 4A). To further characterize the mechanisms enabling tissue-specific transcription, we compared the molecular state of 20E-activated TSSs of primary targets in two tissues—salivary glands and brain—using ChIP-Seqs of Rpb3, TBP, and EcR on control nonheat-shocked *hsp-e23* wandering larvae (Fig. 4B and Supplementary Fig. S11). Surprisingly, most 20E-activated TSSs of primary salivary gland targets retained Rbp3 and EcR peaks in the brain, where they had lower transcriptional levels.

We analysed the molecular states of the 105 20E-activated TSSs in salivary glands by plotting the average binding in both tissues (Fig. 4C). We detected lower Rpb3, TBP and EcR binding levels at the analysed TSSs in the brain compared with salivary glands. The *eip74ef* TSSs retained all the transcriptional proteins analysed in the brain, while having substantially lower transcriptional level in this tissue (Fig. 4D). Thus, the higher transcriptional level of 20E-activated TSSs in salivary glands is partially achieved through promoting Rpb3 recruitment and partially through the control of RNA Pol II promoter escape (Fig. 4E).

These lower Rpb3, TBP, and EcR binding levels observed at 20E-activated TSSs in the brain compared with salivary glands are not explained by overall changes in their binding to the genome; no changes in average Rpb3, TBP, and EcR binding levels were detected when assessing all *Drosophila* TSSs (Supplementary Fig. S12).

Surprisingly, 259 20E-suppressed TSSs in salivary glands had similar numbers of Rpb3, TBP, and EcR binding peaks and levels in brains and salivary glands, with higher transcriptional levels in the brain (Supplementary Fig. S13). This signifies that the increased activity of these promoters in the brain is not through increased Rpb3 recruitment and transcription initiation but via the stimulation of RNA Pol II promoter escape. In addition, this demonstrates that the repressive effect of 20E on these promoters in salivary glands is achieved by the control of RNA Pol II promoter escape.

### E23 expression in the salivary glands suppresses ‘active’ ecdysone-sensitive elements co-bound with EcR and CBP/Nejire at 20E-activated loci

**Figure 5.**
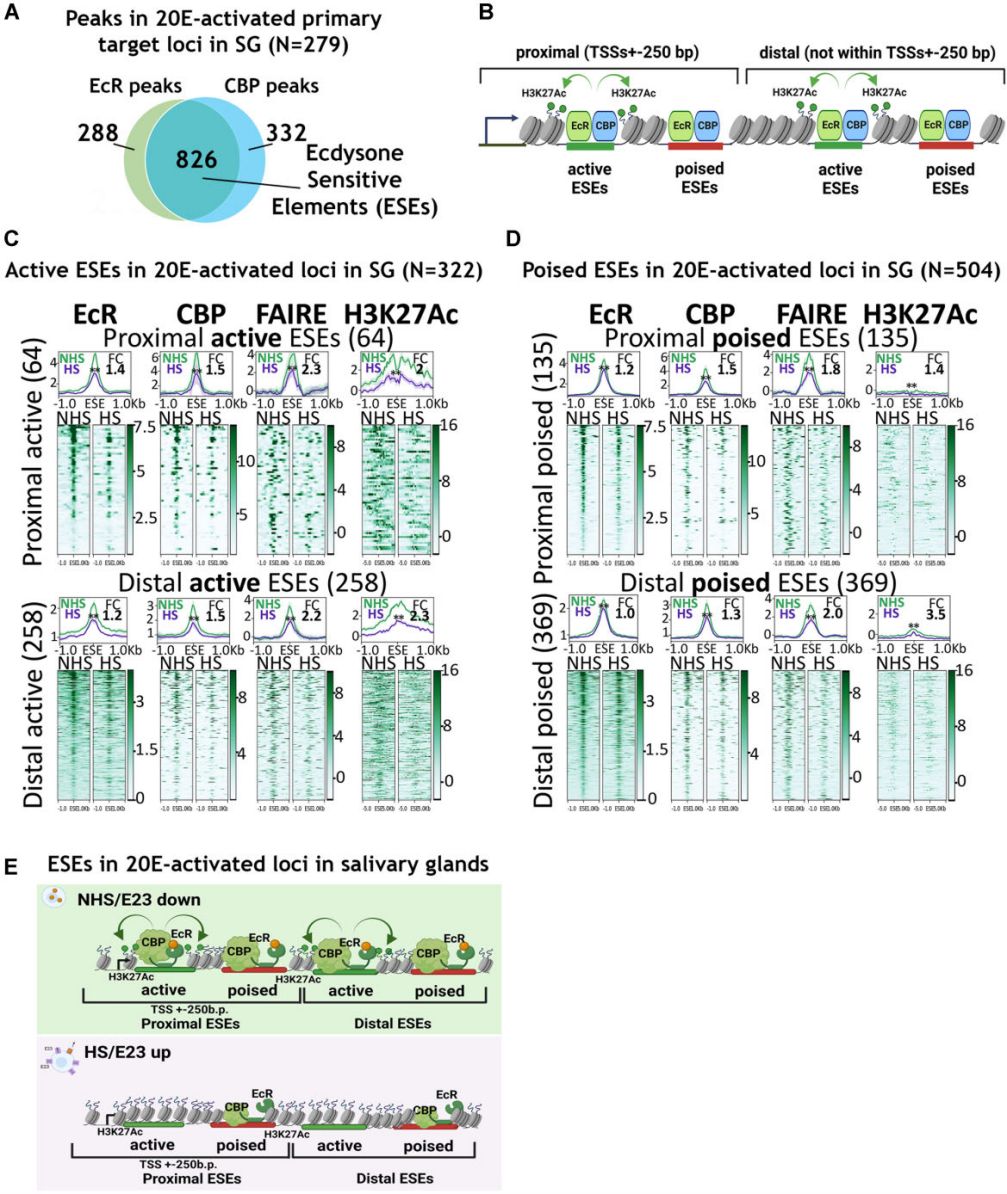
E23 expression in the salivary glands suppresses ‘active’ ESEs co-bound with EcR and CBP/Nejire at 20E-activated loci. (**A**) Venn diagram reflecting an intersection of EcR and CBP/Nejire peaks located in 20E-activated loci ±5 kb from a total number of EcR and CBP/Nejire peaks in these loci defined by ChIP-Seqs in salivary glands of *hsp-e23* wandering larvae in untreated conditions. Regions co-bound with CBP/Nejire and EcR were termed ESEs. (**B**) A scheme displaying two principles of classification of ESEs. Location: ESEs located in ±250 bp of TSSs were defined as proximal ESEs, ESEs located outside of the TSSs but within gene loci, ±5 kb were called distal ESEs. Activity: ESEs are categorized based on their levels of H3K27Ac acetylation. Those with high levels of H3K27Ac are considered ‘active’. ESEs with lower levels of H3K27Ac are ‘poised’. Hierarchical clustering was used to separate ESEs into groups based on their activity levels (provided in Supplementary Fig. S14). Created in BioRender (https://BioRender.com/0ngjjab). (**C**) Average distribution of EcR, CBP, FAIRE, H3K27Ac enrichment estimated by ChIP-Seqs at ‘active’ proximal (located ±250 bp of TSSs, N = 64) and active distal (located outside of the TSSs but within gene loci, ±5 kb, N = 258) ESEs in 20E-activated primary loci in salivary glands in control condition (NHS) and after treatment of *hsp-e23* larvae 20–22 h before pupariation with double 1-h heat shock (with a 1-h rest at RT) (HS). (**D**) Average distribution of EcR, CBP, FAIRE, H3K27Ac proteins estimated by ChIP-Seqs at ‘poised’ proximal (located ±250 bp of TSSs, N = 135) and ‘poised’ distal (located outside of the TSSs but within gene loci, ±5 kb, N = 369) ESEs in 20E-activated primary loci in salivary glands in control condition (NHS) and after treatment of *hsp-e23* larvae 20–22 h before pupariation with double 1-h heat shock (with a 1-h rest at RT) (HS). For panels (C) and (D) ChIP-Seq binding levels were calculated as a ratio to Input (for FAIRE and H3K27Ac the Input was subtracted from sample). The X-axis represents the distance to the ESE in kbp. Average profiles were calculated as a median of binding level with the standard error displayed on the profiles. The FC was calculated using normalized coverage within 500 bp around the summit peak for EcR, CBP, FAIRE and within 1000 bp around the summit peak for H3K27Ac of the analysed ESEs (as a ratio of NHS signal to HS signal). The results of the paired *t*-test analysis are provided on the graphs, where ‘**’ means *P* ≤ .01. (**E**) Scheme displaying that the transcriptional response to 20E in salivary glands involves activating a subset of ESEs with high H3K27Ac levels, highlighting the importance of 20E in regulating chromatin state and EcR binding dynamics. Created in BioRender (https://BioRender.com/n650dzg).

It is widely accepted that 20E activates target transcription by acting through EcR-bound enhancers [12, 46]. To characterize the landscape of enhancers in 20E-activated salivary gland targets, we estimated CBP/Nejire acetyltransferase and EcR-binding sites using ChIP-Seq on salivary gland material obtained from control nonheat-shocked *hsp-e23* wandering larvae. We considered that CBP/Nejire and EcR sites regulated 20E-activated targets if they fell within 20E-activated gene loci, ±5 kb. We observed a strong correlation between CBP/Nejire and EcR binding. Most of the peaks called inside 20E-activated loci possessed both CBP/Nejire and EcR-bound proteins (N = 826; ∼3 co-bound sites per locus on average) (Fig. 5A) (Supplementary Table S14). We considered them important regulatory sites that harbour enhancer properties (due to CBP/Nejire peaks) and are sensitive to 20E (due to EcR peaks); we named them ecdysone-sensitive elements (ESEs).

To determine if 20E is involved in the formation of these ESEs, we compared the binding levels of CBP/Nejire and EcR in the salivary glands of *hsp-e23* larvae expressing E23 subjected to heat shock with those under control conditions without heat shock, as outlined in Fig. 2A. A significant proportion of ESEs had reduced EcR binding according to DiffBind (312 out of 826 peaks), while CBP/Nejire binding was hardly altered (66 out of 826 peaks) (FC ≥ |1|, *P* ≤ .05). In other words, 20E depletion had a much stronger effect on the distribution of EcR in 20E-activated loci than on CBP/Nejire binding. From the total of 279 20E-activated loci, we identified 178 with decreased EcR binding at ESEs, indicating that decrease in EcR binding upon E23 expression occurs at most 20E-activated loci.

For further analysis, we divided ESEs within 20E-activated loci into ‘active’ and ‘poised’ based on the level of H3K27Ac modification inherent to active enhancers (using hierarchical clustering) (Supplementary Fig. S14). Most ESEs in 20E-activated loci were defined as ‘poised’ (N = 504), while the remainder fell into regions with high H3K27Ac levels and were defined as ‘active’ (N = 322) (Supplementary Tables S15–S16). Considering the possible influence of closely located TSSs on the action of ESEs, we further divided the ‘active’ and ‘poised’ ESEs groups into proximal (located ±250 bp of TSSs) and distal (located outside of the TSSs but within gene loci, ±5 kb) for separate analysis (Fig. 5B) (Supplementary Tables S17–S20).

To describe ESE groups, we estimated the enrichment of DNA-binding protein motifs in these regions. The MEME-ChIP tool did not show enrichment of any previously described motifs in proximal ‘active’ or ‘poised’ ESEs (possibly due to the small number of regions used in the analysis) [47]. Distal ‘poised’ ESEs were enriched for Trl/GAF (e-value 3.4e-069), Crol (e-value 5.8e-026), and Aef1 motifs (e-value 9.2e-018) [48–50]. Distal ‘active’ ESEs were enriched for Aef1 (e-value 1.1e-005) and Crp motifs (e-value 2.8–004) [43, 51]. The presence of binding sites for many proteins in the ESEs of 20E-activated loci is consistent with the current view on the cooperative binding of active enhancers by various DNA-binding proteins [52, 53].

To further characterize ESEs groups, we examined chromatin accessibility and protein enrichment at these sites upon 20E depletion (Fig. 5C and D and Supplementary Fig. S15). We observed decreased EcR binding in both proximal and distal ‘active’ ESE groups and stable binding to ‘poised’ ESEs. This shows that dynamic ESEs, where EcR binding is dependent on 20E levels, represent the cohort of ‘active’ ESEs. CBP/Nejire binding levels also decreased upon 20E depletion in ‘active’ ESEs, with a moderate decrease in the ‘poised’ groups. We found a substantial reduction in chromatin accessibility (assessed by FAIRE-Seq analysis) in all ESEs groups tested at 20E-activated loci in response to 20E depletion, demonstrating chromatin state control at these sites by hormone concentration. The chromatin acetylation level (assessed by ChIP-Seq of H3K27Ac) was reduced in both proximal and distal groups of ‘active’ ESEs upon 20E depletion, correlating with the transcriptional suppression of 20E-activated loci (Fig. 5C and D). To quantify changes in chromatin acetylation at ESEs upon 20E depletion, we assessed the level of H3K27Ac enrichment in regions around ESEs (±1 kb) in biological replicates separately and performed paired *t*-test analyses. We observed a significant reduction in H3K27Ac levels in almost half of the ‘active’ ESEs. Thus, 26 of 64 (41%) proximal ‘active’ and 105 of 258 (41%) distal ‘active’ ESEs showed decreased level of chromatin acetylation (FC ≥ |1|, *P* ≤ .05). While in the poised group, the reduction in H3K27Ac level affected only 27 of 135 (20%) proximal and 72 of 369 (20%) distal ESEs. All changes observed in ESEs were specific to 20E-activated loci as there were no substantial changes in the levels of analysed proteins and accessibility at control sites. i.e. all *Drosophila* genome TSSs (Supplementary Fig. S16).

Thus, 20E-activated loci in salivary glands are filled with numerous ESEs, co-bound with EcR and CBP (Fig. 5E). Most of these ESEs represent a ‘poised’ cohort with a low level of the active enhancer marker H3K27Ac. Supposedly, the transcriptional activation of these loci by 20E occurs via a smaller group of ‘active’ ESEs with a high H3K27Ac level (representing one-third of all ESEs). Interestingly, EcR binding at ‘active’ ESEs is 20E-dependent, demonstrating the dynamic nature of these regions (and possible *de novo* EcR recruitment at these sites during development upon an increase in 20E levels). We observed a substantial decrease in chromatin accessibility in all ESEs groups investigated upon 20E depletion, demonstrating the significance of the 20E signal input in chromatin state regulation. The most important finding is that 20E depletion caused a decrease in H3K27Ac in ‘active’ ESEs, which regulates the activity of 20E targets. This finding led us to propose a molecular mechanism for the transcriptional response to 20E in salivary glands. That is, the transcriptional response to 20E is through the activation of a portion of numerous ESEs within target loci.

For a deeper understanding of the influence of 20E depletion on enhancer activity in the genome, we analysed the impact of E23 expression in salivary glands on EcR and CBP/Nejire co-bound sites (ESEs) within 20E-suppressed loci with upregulated transcription after heat shock (Supplementary Fig. S17). To this end, we called EcR and CBP/Nejire peaks using ChIP-Seqs under both heat-shocked and nonheat shocked conditions, expressing E23, and selected the co-bound sites, i.e. ESEs (N = 637) (Supplementary Table S21). We found a small number of downregulated ESEs in 20E-suppressed loci upon E23 expression. At the same time, a significant proportion of sites exhibited an increase in EcR and CBP binding according to DiffBind (152 and 105 for EcR, and 50 and 80 for CBP, upregulated and acquired, respectively). To analyse the average binding, we divided ESEs of 20E-suppressed loci into two groups: proximal (within ±250 bp of TSSs) and distal (outside of the TSSs but within gene loci ±5 kb) (Supplementary Tables S22 and S23). We observed an increase in EcR and CBP binding both in proximal and distal ESEs, along with a substantial increase in H3K27Ac mark levels (especially at distal ESEs). The tracks at individual loci align with our observations from the average graphs both for 20E-activated and 20E-suppressed loci in the salivary glands (Supplementary Fig. S18).

Changes in EcR binding at 20Е-activated and 20E-suppressed loci upon 20E depletion illustrate the dependence of the binding process on 20E concentration. We observed a correlation between the presence of EcR at loci and the activity of those loci. The recruitment of EcR to 20E-suppressed loci in the 20E-depleted state challenges the established view of unliganded EcR as an active repressor. We propose that the observed precedent of positive transcriptional regulation by unliganded EcR may be intrinsic to salivary glands. It is known that the EcR-B1 isoform predominates in salivary glands prior to metamorphosis and rescues the puffing phenotype of polytene chromosomes [54, 55]. The EcR-B1 isoform contains a ligand-independent activator domain at the N-terminus, which has been demonstrated to activate transcription in reporter assays in the absence of 20E [56–58]. Thus, the active expression of 20E-suppressed genes following EcR recruitment to their loci upon 20E depletion may be attributable to this specific property of the EcR-B1 isoform present in salivary glands. Regarding a possible mechanism that explains the redistribution of EcR between sites in the genome in the context of 20E depletion, we can hypothesize that 20E exerts opposing effects on the affinity of EcR for various DNA-binding proteins that facilitate its recruitment to different types of enhancers. In this context, elucidating new interactions between EcR and DNA-binding proteins that are dependent on hormone concentration would be of great interest. An alternative mechanism to explain the increase in EcR affinity for some sites in the 20E-depleted state could be competition of EcR for these sites at high 20E titer with other nuclear receptors whose transcription is induced by 20E as part of the ecdysone cascade (similar to the previously described mechanism of competition of ER with the nuclear receptors GR or AR for EREs under high titer of E2) [59–61].

### ‘Active’ ESEs, sensitive to 20E depletion, are tissue-specific, as evidenced by our and previously published data

Using RNA-seq data, we demonstrated the tissue-specific transcriptional pattern of 20E-activated and 20E-suppressed targets in the salivary glands (Fig. 2F and G). In this section, we considered whether the state of ‘active’ ESEs at these gene loci is also tissue-specific.

**Figure 6.**
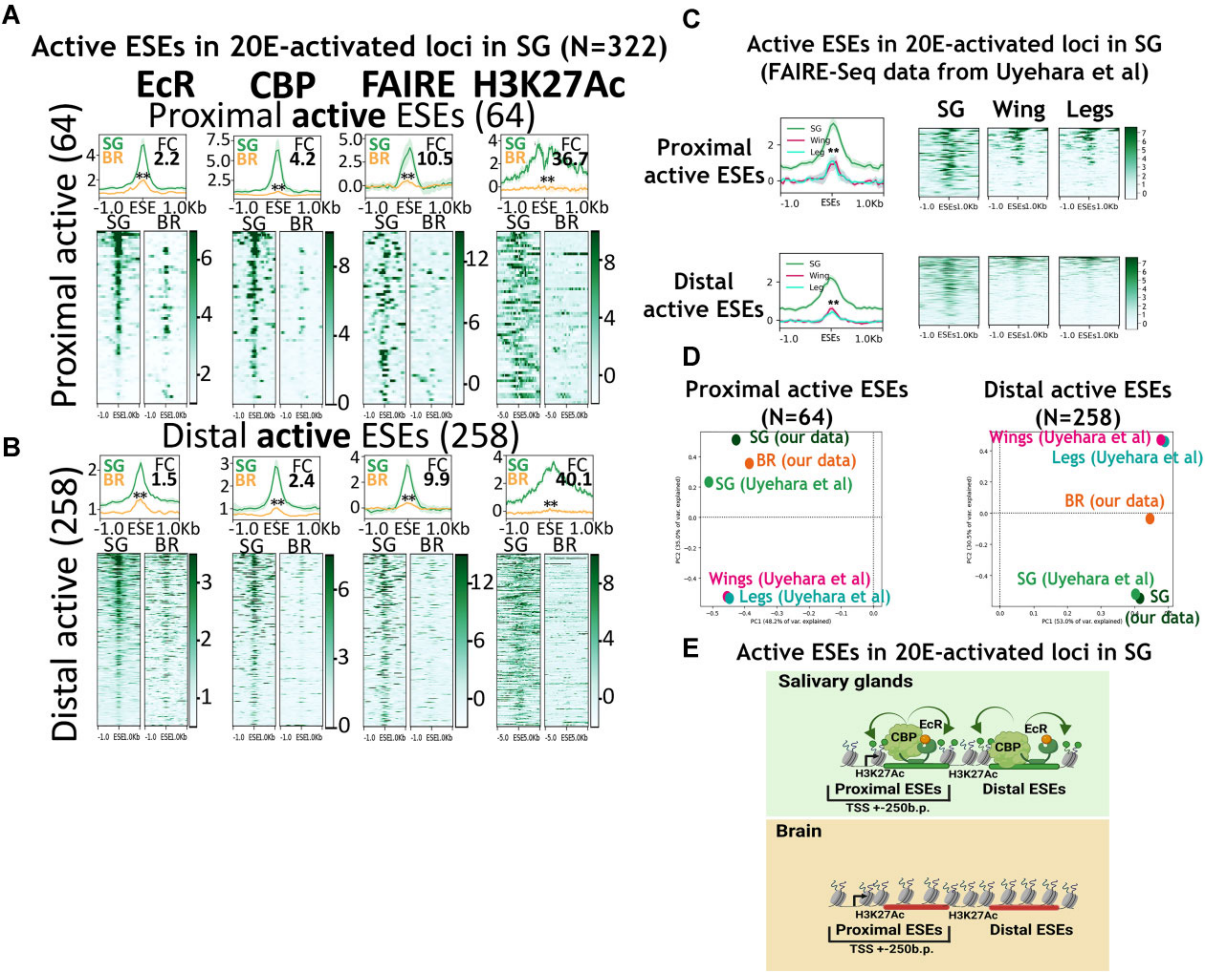
‘Active’ ESEs, sensitive to 20E depletion, are tissue-specific, as evidenced by our and previously published data. Average distribution of EcR, CBP, FAIRE and H3K27Ac enrichment estimated by ChIP-Seqs at (**A**) ‘active’ proximal (located ±250 bp of TSSs, N = 64) ESEs and (**B**) ‘active’ distal (located outside of the TSSs but within gene loci, ±5 kb, N = 258) ESEs in salivary glands (SG) and brain (BR) of *hsp-e23* wandering larva in untreated conditions. ChIP-Seq binding level was calculated as a ratio to Input. For FAIRE-Seq and H3K27Ac the Input was subtracted from sample. The X-axis represents the distance to the ESE in kbp. Average profiles were calculated as a median of binding level with the standard error displayed on the profiles. The FC was calculated using normalized coverage within 500 bp around the summit peak for EcR, CBP, FAIRE and within 1000 bp around the summit peak for H3K27Ac of the analysed ESEs (as a ratio of SG signal to a BR signal). The results of the paired *t*-test analysis are provided on the graphs, where ‘**’ means *P* ≤ .01. (**C**) Average FAIRE-Seq signals from the previously published data (from [14]) at distal (N = 258) and proximal (N = 64) ‘active’ ESEs of 20E-activated targets in salivary glands. The FC was calculated using normalized coverage within 500 bp around the summit peak of the analysed ESEs (as a ratio of SG signal to Wing and Leg signals). The FC for proximal ‘active’ loci were 5.1 for SG/Wing and 4.0 for SG/Leg. The FC for distal ‘active’ loci were 9.7 for SG/Wing and 13.2 for SG/Leg. The results of the paired *t*-test analysis are provided on the graphs, where ‘**’ means *P* ≤ .01. (**D**) PCA plot represents a strong correlation between the FAIRE-Seq signals for the salivary glands obtained previously and in a current study. In other tissues the chromatin accessibility levels at the described ESEs varied. For the PCA analysis FAIRE-Seq signals were calculated using normalized coverage within 500 bp around the summit peak of the analysed ESEs. (**E**) Model illustrating the regulation of 20E-activated target loci in salivary glands. We suggest that 20E-activated targets are regulated by a tissue-specific set of ‘active’ ESEs. In salivary glands, these ‘active’ ESEs have CBP/Nejire and EcR peaks, as well as increased chromatin accessibility and acetylation, whereas in the brain, they are silent. Created in BioRender (https://BioRender.com/okxy4sl).

We assessed the molecular state of proximal and distal ‘active’ ESEs in 20E-activated targets in the salivary glands in two tissues (Fig. 6A and B, and Supplementary Figs S19 and S20). We found evidence of tissue-specific activity in ‘active’ ESEs groups in the salivary glands compared with the brain. CBP/Nejire and EcR were observed at all ‘active’ ESEs in 20E-activated targets in salivary glands but not in the brain. Chromatin accessibility and acetylation were consistent with CBP/Nejire and EcR binding. So, our data indicate that 20E-activated target genes in salivary glands are controlled by a tissue-specific pool of ‘active’ ESEs. Furthermore, ‘active’ ESEs possess the characteristics of completely silent enhancers in the brain, lacking CBP/Nejire binding and with decreased chromatin accessibility.

Since chromatin accessibility at the enhancers is strongly correlated with their activity in a particular tissue, we reviewed data previously published by Uyehara *et al.* to also characterize the state of ‘active’ ESEs in other tissues [14]. Using the same stage of Drosophila development allowed us to conduct this analysis (Fig. 6C). We estimated the average FAIRE-Seq signal at proximal and distal ‘active’ ESEs in 20E-activated loci in the salivary glands using the data obtained by Uyehara *et al.* for salivary glands, wing discs, and leg discs of wandering larvae. Both proximal and distal ‘active’ ESEs exhibited increased chromatin accessibility in salivary glands compared with other tissues, consistent with our data. A correlation analysis of FAIRE-Seq signal enrichment at ‘active’ ESEs using both our and previously published datasets (Fig. 6D) revealed a strong correlation between these datasets regarding the salivary glands. In other tissues, chromatin accessibility levels at the described ESEs were different. This observation strongly supports our data regarding the tissue specificity of ‘active’ ESEs in 20E-activated targets in salivary glands. Importantly, our data align with those of previously published research.

As shown in previous sections, 20E-suppressed loci in salivary glands demonstrated tissue-specific transcriptional patterns, which were opposite to 20E-activated targets; 20E-suppressed loci had brain-enriched transcription (Fig. 2G). We conducted a comparative analysis of proximal and distal ESEs (N = 637), co-bound with EcR and CBP/Nejire in 20E-suppressed salivary gland targets, in the salivary glands and brain (Supplementary Fig. S21). Supplementary Fig. S17 shows that these ESEs possess EcR and CBP/Nejire binding in salivary glands under control conditions and upon E23 expression, but exhibit increased H3K27Ac modifications under heat-shocked conditions. Remarkably, we observed a ‘tissue-specific’ state for these ESEs in 20E-suppressed loci, which was in apparent contrast to the transcriptional activity of their loci (Supplementary Fig. S21). These ESEs demonstrated decreased CBP/Nejire binding as well as chromatin accessibility in the brain where their genes loci were found to be transcriptionally upregulated. Considering that H3K27Ac levels at these ESEs in salivary glands were relatively low, we suggest that they persisted in this tissue in a ‘poised’ state. Altogether, this means that transcriptional suppression by 20E at 20E-suppressed loci is mediated through salivary gland-specific ESEs, while transcription of these genes in the brain is regulated by other groups of enhancers. Indeed, CBP/Nejire ChIP-Seq in the brain revealed a group of brain-specific CBP/Nejire binding sites that had increased chromatin accessibility (Supplementary Fig. S22) (Supplementary Table S24).

## Discussion

### E23 expression causes an impaired ecdysone-signalling phenotype and reduces the transcription of well-known 20E targets in *Drosophila* salivary glands

The opposing effects of 20E in larval and adult tissues during *Drosophila* metamorphosis provide a convenient model for studying the tissue-specific effects of steroids. Unlike mammalian steroids, 20E is a major steroid hormone in *Drosophila*, controlling various physiological transitions [62]. The role of steroids in gene transcription is easier to describe in a simple *Drosophila* system.

To study the effects of 20E on tissues, it is first necessary to characterize the target genes. In *Drosophila*, this stage is complicated as the concentration of 20E peaks many times during development. At each stage, even in the same tissue, 20E activates various targets. Thus, at the wandering larval stage, 20E activates cell death genes in the fat body, while the salivary glands survive this 20E peak without signs of destruction [63]. Activation of cell death targets in the salivary glands occurs during the second prepupal 20E pulse [64]. 20E in the salivary glands of the third larval and prepupa stages appears to affect different pools of genes.

There are two main approaches towards identifying 20E target genes in tissues. The first is to study transcriptional profiles in knockout/knockdown flies with decreased EcR expression. The second is to employ *ex vivo* tissue culture in a medium containing 20E. Both methods have their limitations. The use of knockouts and knockdowns leads to the description of targets affected by 20E together with the EcR targets it controls in the absence of the hormone [11, 13]. Organ culture in a medium containing 20E allows for more specific target identification; however, it often does not lead to a full level of target activation, resulting in the identification of a depleted target pool [65].

In our work, we employed a third approach—expression in *Drosophila* tissues of the membrane transporter E23, which exports 20E from the cell. We believe that this approach complements and helps to solve the challenges of identifying 20E targets using the two aforementioned methods. Artificial E23 expression before the first 20E pulse in metamorphosis revealed a large number of target genes (N = 637) in the salivary glands dependent on the 20E concentration. However, E23 expression in the brain at this stage only mildly affected transcription. This was not due to the absence of the artificial E23 protein as its presence was confirmed by western blot, although at lower levels. The resistance of brain tissue to E23 at this developmental stage was due to the total lack of ecdysone-dependent transcriptional induction. This was evident from the lower transcriptional levels of previously known 20E-dependent targets in the brain compared with salivary glands. This was indirectly confirmed in a study by Uyehara *et al.* They reported that, at the wandering larval stage, there was no ecdysone cascade gene expression in another adult fly tissue—wing imaginal discs [14]. We do not yet have a firm explanation for why 20E fails to activate the transcription of its target genes in the brain at the wandering larval stage. We believe this may be due to the more effective repression of 20E targets in brain tissue. We found that some signalling pathways opposing ecdysone signalling were more active in the brain than in the salivary glands, e.g. *dpp* [66]. According to our RNA-seq data, the main *dpp* signalling components were transcribed in the brain at higher levels than in the salivary glands (Supplementary Fig. S23). It is likely that the difference in tissue response to 20E is also related to their enrichment with different EcR isoforms [54, 55, 67]. Perhaps the presence of the EcR-A isoform in adult tissues, which has repressive properties, prevents them from being activated by 20E during the first metamorphosis pulse.

The effect of E23 expression on gene transcription in the salivary glands was as expected. We observed a decrease in the transcription of many ecdysone cascade genes as well as *sgs* genes, whose action is controlled by 20E. The 20E targets identified were present in pools of previously described 20E targets, obtained using EcR knockdown and by culturing various *Drosophila* cell lines with 20E (Supplementary Fig. S24) [14, 68].

### E23 expression and 20E depletion impair Pol II and TBP recruitment at the promoters of 20E-activated genes in the salivary glands of wandering larvae

Promoters are the end point of all transcriptional regulatory signals; their state necessarily changes during transcriptional activation/repression. In our previous research on cultured cells, we showed that some 20E cascade genes are activated by the hormone through the transcription elongation stage and harbour Pol II bound to their promoters in their inactive state [16]. We demonstrated a physical interaction between EcR and NELF, the best-known protein complex responsible for regulating transcriptional elongation, and its involvement in the active transcriptional phase of 20E-activated genes [17]. In the current manuscript, for the first time, we have studied the molecular state of *Drosophila* tissue promoters upon 20E depletion. We demonstrated that 20E depletion, through E23 expression, substantially decreases Pol II levels at the promoters of 20E-activated genes in the salivary glands; i.e. affecting the transcription initiation stage. A concomitant decrease in TBP binding indicates that the Pol II recruitment issues upon 20E depletion may be a result of PIC defects. This finding, together with the observed direct binding of EcR to the promoters of the regulated genes, suggests a possible direct connection between EcR and the machinery enabling transcription initiation; this has not been reported to date. The decrease in TBP binding upon 20E depletion suggests a possible EcR interaction with the TFIID subunit of the PIC and the promotion of its binding upon an increase in hormone levels. However, a previously reported direct interaction between EcR and the RPII215 subunit may indicate the possibility of direct Pol II recruitment stimulation [15]. Combining existing and novel data, we suggest an influence of 20E on various transcriptional stages during the activation of its target promoters in tissues. We propose that promoters may react differently to low and high 20E concentrations during development; the former may stimulate Pol II recruitment and maintain promoters in a ‘ready-to-be-induced’ state, while the latter may stimulate the elongation stage (which could lead to a two-phase gene activation mechanism) [69]. Considering the experimental difficulties involved in studying these mechanisms, it would be more appropriate to investigate them using individual 20E-dependent model genes.

### 20E depletion upon E23 expression decreases chromatin accessibility and acetylation at active ESEs of its targets in the salivary glands

EcR is a nuclear receptor known to bind enhancers of its target loci and activate them upon interaction with 20E [46, 62]. By analysing the loci of 20E-activated genes in the salivary glands, we found that they possessed many EcR and CBP/Nejire acetyltransferase co-bound sites, which we named ESEs. The active state of a substantial part of these ESEs was dependent on high 20E concentrations; they lost EcR and CBP/Nejire binding and exhibited decreased chromatin accessibility and acetylation upon E23 expression. We concluded that the transcriptional control of 20E-activated genes is exerted through the ‘active’ ESEs, whose activity is completely dependent on the concentration of 20E. This dependency begins with their accessibility to transcription factor and coregulator binding and ends with their CBP/Nejire enhancer-specific acetyltransferase activity.

Our results may seem to contradict previous findings on the effect of 20E on chromatin state. Uyehara *et al.*, recently showed that EcR knockdown did not affect chromatin accessibility at the enhancers of wing imaginal discs [14]. However, these differences can be reconciled. Uyehara *et al.* examined enhancers in the wing discs of wandering larvae, where EcR knockdown had a minimal impact on transcription according to their data. Presumably, at the wandering larval stage, an increase in 20E has a greater effect on the salivary glands than on adult tissues. In the current study, we attributed the finding of ESEs, where 20E controls chromatin accessibility and acetylation, to the salivary gland’s high sensitivity to 20E (which was observed in both studies by RNA-seq experiments). We propose that EcR knockdown would affect enhancers in the salivary glands similarly to E23 expression.

How does 20E affect chromatin accessibility in the salivary glands? Primary transcriptional activation by 20E in cultured cells was strongly dependent on two coactivators affecting chromatin structure—Brahma (SWI/SNF component) and CBP/Nejire [16]. At the same time, other chromatin remodellers had the opposite effect—knockdown of Mi-2 and CHD1 leads to stronger transcriptional responses upon induction by 20E. That is, the influence of Mi-2 and CHD1 remodellers is rather restricting [16, 70]. We did not study the effect of SWI/SNF knockdown on 20E-dependent activation in this work; however, we believe that it is a very promising line for future research. The transcription of individual 20E-dependent genes was previously found to be affected due to mutations in the SWI/SNF subunits [71–73]. However, there has been no genome-wide description of the role of SWI/SNF in the activation of 20E-dependent targets, especially comparing different tissues.

In this study, we examined the impact of 20E depletion on the recruitment of the CBP/Nejire acetyltransferase, a key coregulator in the 20E response [16, 74, 75]. We observed reduced CBP/Nejire binding at 20E-dependent ESEs where 20E alters chromatin accessibility upon the depletion. The causal relationship between CBP/Nejire recruitment and chromatin decompaction remains unclear; recruitment could either result from or cause increased chromatin accessibility. Further investigation, perhaps through the use of specific inhibitors, might clarify the role of CBP/Nejire in chromatin modifications at enhancers involved in the 20E response [76, 77].

An important finding of the current study is the dynamic nature of EcR binding to its target loci. We observed a change in EcR binding following 20E depletion: specifically, an increase in binding at 20E-activated loci and a decrease at 20E-supressed loci. This change in EcR binding was evident in both the number of bound peaks and the quantity of bound protein. Notably, several ESEs remained associated with 20E-dependent target loci both prior to and following 20E depletion. It appears that variations in 20E titer did not affect the overall affinity of EcR for target loci, but rather altered its capacity to spread within these loci, thereby exposing additional ESEs. It is hypothesized that these additional sites work synergistically with stable ESEs, facilitating the attainment of the requisite number of active enhancers necessary for transcriptional activation. This mechanism of ESEs cooperativity aligns with the established functions of developmental enhancers and warrants further investigation in future research [53].

## Supplementary Material

gkaf284_Supplemental_Files

## Data Availability

All obtained RNA-seq, ChIP-Seq, FAIRE-Seq data were deposited into the Gene Expression Omnibus – GSE260939 and provided as a UCSC session (https://genome.ucsc.edu/s/VorobyevaNadezhda/Transcriptional%20response%20to%2020E%20in%20tissues). Regions analysed in a manuscript are provided in the Supplementary section.

## References

[1] Martinkovich S, Shah D, Planey SL et al. Selective estrogen receptor modulators: tissue specificity and clinical utility. Clin Interv Aging. 2014; 9:1437–52. 25210448 10.2147/CIA.S66690 PMC4154886

[2] Pihlajamaa P, Sahu B, Jänne OA Determinants of receptor- and tissue-specific actions in androgen signaling. Endocr Rev. 2015; 36:357–84. 10.1210/er.2015-1034. 26052734

[3] Kozlova T, Thummel CS Steroid regulation of postembryonic development and reproduction in Drosophila. Trends Endocrinol Metab. 2000; 11:276–80. 10920384 10.1016/s1043-2760(00)00282-4

[4] Thummel CS Molecular mechanisms of developmental timing in *C. ele**gan**s* and drosophila. Dev Cell. 2001; 1:453–65. 10.1016/S1534-5807(01)00060-0. 11703937

[5] Zhimulev IF, Belyaeva ES, Semeshin VF et al. Polytene chromosomes: 70 years of genetic research. Int Rev Cytol. 2004; 241:203–75. 15548421 10.1016/S0074-7696(04)41004-3

[6] Yin VP, Thummel CS Mechanisms of steroid-triggered programmed cell death in Drosophila. Semin Cell Dev Biol. 2005; 16:237–43. 10.1016/j.semcdb.2004.12.007. 15797834

[7] Beira JV, Paro R The legacy of Drosophila imaginal discs. Chromosoma. 2016; 125:573–92. 10.1007/s00412-016-0595-4. 27153833 PMC5023726

[8] Bonneton F, Laudet V. Gilbert LI 6 - Evolution of nuclear receptors in insects. Insect Endocrinology. 2012; San Diego Academic Press 219–52. 10.1016/B978-0-12-384749-2.10006-8.

[9] Koelle MR, Talbot WS, Segraves WA et al. The drosophila EcR gene encodes an ecdysone receptor, a new member of the steroid receptor superfamily. Cell. 1991; 67:59–77. 1913820 10.1016/0092-8674(91)90572-g

[10] Hill RJ, Billas IML, Bonneton F et al. Ecdysone receptors: from the Ashburner model to structural biology. Annu Rev Entomol. 2013; 58:251–71. 23072463 10.1146/annurev-ento-120811-153610

[11] Krasnov AN, Evdokimova AA, Mazina MY et al. Coregulators reside within Drosophila ecdysone-inducible loci before and after ecdysone treatment. Int J Mol Sci. 2023; 24:11844 10.3390/ijms241411844. 37511602 PMC10380596

[12] Shlyueva D, Stelzer C, Gerlach D et al. Hormone-responsive enhancer-activity maps reveal predictive motifs, indirect repression, and targeting of closed chromatin. Mol Cell. 2014; 54:180–92. 10.1016/j.molcel.2014.02.026. 24685159

[13] Johnston DM, Sedkov Y, Petruk S et al. Ecdysone- and NO-mediated gene regulation by competing EcR/usp and E75A nuclear receptors during Drosophila development. Mol Cell. 2011; 44:51–61. 10.1016/j.molcel.2011.07.033. 21981918 PMC3190167

[14] Uyehara CM, Leatham-Jensen M, McKay DJ Opportunistic binding of EcR to open chromatin drives tissue-specific developmental responses. Proc Natl Acad Sci USA. 2022; 119:e2208935119 10.1073/pnas.2208935119. 36161884 PMC9546573

[15] Mazina MY, Ziganshin RH, Magnitov MD et al. Proximity-dependent biotin labelling reveals CP190 as an EcR/Usp molecular partner. Sci Rep. 2020; 10:4793 10.1038/s41598-020-61514-0. 32179799 PMC7075897

[16] Mazina MY, Kovalenko EV, Derevyanko PK et al. One signal stimulates different transcriptional activation mechanisms. Biochim Biophys Acta. 2018; 1861:178–89. 10.1016/j.bbagrm.2018.01.016. 29410380

[17] Mazina MY, Kovalenko EV, Vorobyeva NE The negative elongation factor NELF promotes induced transcriptional response of Drosophila ecdysone-dependent genes. Sci Rep. 2021; 11:172 10.1038/s41598-020-80650-1. 33420323 PMC7794308

[18] Hock T, Cottrill T, Keegan J et al. The E23 early gene of Drosophila encodes an ecdysone-inducible ATP-binding cassette transporter capable of repressing ecdysone-mediated gene activation. Proc Natl Acad Sci USA. 2000; 97:9519–24. 10.1073/pnas.160271797. 10931948 PMC16897

[19] Itoh TQ, Tanimura T, Matsumoto A Membrane-bound transporter controls the circadian transcription of clock genes in Drosophila. Genes Cells. 2011; 16:1159–67. 10.1111/j.1365-2443.2011.01559.x. 22077638

[20] Erokhin M, Gorbenko F, Lomaev D et al. Boundaries potentiate polycomb response element-mediated silencing. BMC Biol. 2021; 19:113 10.1186/s12915-021-01047-8. 34078365 PMC8170967

[21] Vorobyeva NE, Soshnikova NV, Nikolenko JV et al. Transcription coactivator SAYP combines chromatin remodeler Brahma and transcription initiation factor TFIID into a single supercomplex. Proc Natl Acad Sci USA. 2009; 106:11049–54. 10.1073/pnas.0901801106. 19541607 PMC2708721

[22] Markstein M, Pitsouli C, Villalta C et al. Exploiting position effects and the gypsy retrovirus insulator to engineer precisely expressed transgenes. Nat Genet. 2008; 40:476–83. 10.1038/ng.101. 18311141 PMC2330261

[23] Rank G, Prestel M, Paro R Transcription through intergenic chromosomal memory elements of the drosophila bithorax complex correlates with an epigenetic switch. Mol Cell Biol. 2002; 22:8026–34. 10.1128/MCB.22.22.8026-8034.2002. 12391168 PMC134728

[24] Kovalenko EV, Mazina MY, Krasnov AN et al. The Drosophila nuclear receptors EcR and ERR jointly regulate the expression of genes involved in carbohydrate metabolism. Insect Biochem Mol Biol. 2019; 112:103184 10.1016/j.ibmb.2019.103184. 31295549

[25] Vorobyeva NE, Nikolenko JV, Nabirochkina EN et al. SAYP and Brahma are important for ‘repressive’ and ‘transient’ Pol II pausing. Nucleic Acids Res. 2012; 40:7319–31. 10.1093/nar/gks472. 22638575 PMC3424582

[26] Mazina MY, Derevyanko PK, Kocheryzhkina EV et al. Coactivator complexes participate in different stages of the *Drosophila melanogaster* hsp70 gene transcription. Russ J Genet. 2017; 2:178–86. 10.1134/S1022795417010094. 29372961

[27] Thummel CS Ecdysone-regulated puff genes 2000. Insect Biochem Mol Biol. 2002; 32:113–20. 10.1016/S0965-1748(01)00112-6. 11755052

[28] Ashburner M, Richards G Sequential gene activation by ecdysone in polytene chromosomes of Drosophila melanogaster,: III. Consequences of ecdysone withdrawal. Dev Biol. 1976; 54:241–55. 10.1016/0012-1606(76)90302-X. 825403

[29] Beckstead RB, Lam G, Thummel CS The genomic response to 20-hydroxyecdysone at the onset of Drosophila metamorphosis. Genome Biol. 2005; 6:R99 10.1186/gb-2005-6-12-r99. 16356271 PMC1414087

[30] Woodard CT, Baehrecke EH, Thummel CS A molecular mechanism for the stage specificity of the Drosophila prepupal genetic response to ecdysone. Cell. 1994; 79:607–15. 10.1016/0092-8674(94)90546-0. 7954827

[31] Mazina MY, Nikolenko JV, Fursova NA et al. Early-late genes of the ecdysone cascade as models for transcriptional studies. Cell Cycle. 2015; 14:3593–601. 10.1080/15384101.2015.1100772. 26506480 PMC4825724

[32] Terashima J, Takaki K, Sakurai S et al. Nutritional status affects 20-hydroxyecdysone concentration and progression of oogenesis in *Drosophila melanogaster*. J Endocrinol. 2005; 187:69–79. 10.1677/joe.1.06220. 16214942

[33] Ables ET, Hwang GH, Finger DS et al. A genetic mosaic screen reveals ecdysone-responsive genes regulating drosophila oogenesis. G3 (Bethesda). 2016; 6:2629–42. 10.1534/g3.116.028951. 27226164 PMC4978916

[34] Domanitskaya E, Anllo L, Schüpbach T Phantom, a cytochrome P450 enzyme essential for ecdysone biosynthesis, plays a critical role in the control of border cell migration in Drosophila. Dev Biol. 2014; 386:408–18. 10.1016/j.ydbio.2013.12.013. 24373956 PMC4028970

[35] Lehmann M, Wattler F, Korge G Two new regulatory elements controlling the Drosophila Sgs-3 gene are potential ecdysone receptor and fork head binding sites. Mech Dev. 1997; 62:15–27. 10.1016/S0925-4773(96)00644-2. 9106163

[36] Zhang C, Robinson BS, Xu W et al. The ecdysone receptor coactivator Taiman links Yorkie to transcriptional control of germline stem cell factors in somatic tissue. Dev Cell. 2015; 34:168–80. 10.1016/j.devcel.2015.05.010. 26143992 PMC4519380

[37] Bai J, Uehara Y, Montell DJ Regulation of invasive cell behavior by Taiman, a drosophila protein related to AIB1, a steroid receptor coactivator amplified in breast cancer. Cell. 2000; 103:1047–58. 10.1016/S0092-8674(00)00208-7. 11163181

[38] Strassburger K, Lutz M, Müller S et al. Ecdysone regulates Drosophila wing disc size via a TORC1 dependent mechanism. Nat Commun. 2021; 12:6684 10.1038/s41467-021-26780-0. 34795214 PMC8602387

[39] Homem CCF, Steinmann V, Burkard TR et al. Ecdysone and mediator change energy metabolism to terminate proliferation in drosophila neural stem cells. Cell. 2014; 158:874–88. 10.1016/j.cell.2014.06.024. 25126791

[40] Ross-Innes CS, Stark R, Teschendorff AE et al. Differential oestrogen receptor binding is associated with clinical outcome in breast cancer. Nature. 2012; 481:389–93. 10.1038/nature10730. 22217937 PMC3272464

[41] Adato O, Sloutskin A, Komemi H et al. ElemeNT 2023: an enhanced tool for detection and curation of core promoter elements. Bioinformatics. 2024; 40:btae110 10.1093/bioinformatics/btae110. 38407414 PMC10950481

[42] Serebreni L, Pleyer L, Haberle V et al. Functionally distinct promoter classes initiate transcription via different mechanisms reflected in focused versus dispersed initiation patterns. EMBO J. 2023; 42:e113519 10.15252/embj.2023113519. 37013908 PMC10183819

[43] Zhimulev I, Vatolina T, Levitsky V et al. Developmental and housekeeping genes: two types of genetic organization in the drosophila genome. Int J Mol Sci. 2024; 25:4068 10.3390/ijms25074068. 38612878 PMC11012173

[44] Vo Ngoc L, Kassavetis GA, Kadonaga JT The RNA polymerase II core promoter in drosophila. Genetics. 2019; 212:13–24. 10.1534/genetics.119.302021. 31053615 PMC6499525

[45] Qi Z, Jung C, Bandilla P et al. Large-scale analysis of drosophila core promoter function using synthetic promoters. Mol Syst Biol. 2022; 18:e9816 10.15252/msb.20209816. 35156763 PMC8842121

[46] Truman JW The evolution of insect metamorphosis. Curr Biol. 2019; 29:R1252–68. 10.1016/j.cub.2019.10.009. 31794762

[47] Machanick P, Bailey TL MEME-ChIP: motif analysis of large DNA datasets. Bioinformatics. 2011; 27:1696–7. 10.1093/bioinformatics/btr189. 21486936 PMC3106185

[48] Brodu V, Mugat B, Fichelson P et al. A UAS site substitution approach to the in vivo dissection of promoters: interplay between the GATAb activator and the AEF-1 repressor at a Drosophila ecdysone response unit. Development. 2001; 128:2593–602. 10.1242/dev.128.13.2593. 11493575

[49] Chetverina D, Erokhin M, Schedl P GAGA factor: a multifunctional pioneering chromatin protein. Cell Mol Life Sci. 2021; 78:4125–41. 10.1007/s00018-021-03776-z. 33528710 PMC8815297

[50] Erokhin M, Brown JL, Lomaev D et al. Crol contributes to PRE-mediated repression and polycomb group proteins recruitment in Drosophila. Nucleic Acids Res. 2023; 51:6087–100. 10.1093/nar/gkad336. 37140047 PMC10325914

[51] Chetverina D, Vorobyeva NE, Mazina MY et al. Comparative interactome analysis of the PRE DNA-binding factors: purification of the Combgap-, Zeste-, Psq-, and Adf1-associated proteins. Cell Mol Life Sci. 2022; 79:353 10.1007/s00018-022-04383-2. 35676368 PMC11072172

[52] Rao S, Ahmad K, Ramachandran S Cooperative binding between distant transcription factors is a hallmark of active enhancers. Mol Cell. 2021; 81:1651–65. 10.1016/j.molcel.2021.02.014. 33705711 PMC8052300

[53] Loubiere V, de Almeida BP, Pagani M et al. Developmental and housekeeping transcriptional programs display distinct modes of enhancer-enhancer cooperativity in Drosophila. Nat Commun. 2024; 15:8584 10.1038/s41467-024-52921-2. 39362902 PMC11450171

[54] Talbot WS, Swyryd EA, Hogness DS Drosophila tissues with different metamorphic responses to ecdysone express different ecdysone receptor isoforms. Cell. 1993; 73:1323–37. 10.1016/0092-8674(93)90359-X. 8324824

[55] Bender M, Imam FB, Talbot WS et al. Drosophila ecdysone receptor mutations reveal functional differences among receptor isoforms. Cell. 1997; 91:777–88. 10.1016/S0092-8674(00)80466-3. 9413987

[56] Truman JW, Riddiford LM Drosophila postembryonic nervous system development: a model for the endocrine control of development. Genetics. 2023; 223:iyac184 10.1093/genetics/iyac184. 36645270 PMC9991519

[57] Hu X, Cherbas L, Cherbas P Transcription activation by the ecdysone receptor (EcR/USP): identification of activation functions. Mol Endocrinol. 2003; 17:716–31. 10.1210/me.2002-0287. 12554759

[58] Mouillet J-F, Henrich VC, Lezzi M et al. Differential control of gene activity by isoforms A, B1 and B2 of the Drosophila ecdysone receptor. Eur J Biochem. 2001; 268:1811–9. 10.1046/j.1432-1327.2001.02051.x. 11248701

[59] Sun G, Zhao C, Han J et al. Regulatory mechanisms of steroid hormone receptors on gene transcription through chromatin interaction and enhancer reprogramming. Cell Oncol. 2024; 47:2073–90. 10.1007/s13402-024-01011-y. PMC12974005 39543064

[60] Karmakar S, Jin Y, Nagaich AK Interaction of glucocorticoid receptor (GR) with estrogen receptor (ER) α and activator protein 1 (AP1) in dexamethasone-mediated interference of erα activity *. J Biol Chem. 2013; 288:24020–34. 10.1074/jbc.M113.473819. 23814048 PMC3745347

[61] Tonsing-Carter E, Hernandez KM, Kim CR et al. Glucocorticoid receptor modulation decreases ER-positive breast cancer cell proliferation and suppresses wild-type and mutant ER chromatin association. Breast Cancer Res. 2019; 21:82 10.1186/s13058-019-1164-6. 31340854 PMC6651939

[62] Yamanaka N, Rewitz KF, O’Connor MB Ecdysone control of developmental transitions: lessons from Drosophila research. Annu Rev Entomol. 2013; 58:497–516. 10.1146/annurev-ento-120811-153608. 23072462 PMC4060523

[63] Rusten TE, Lindmo K, Juhász G et al. Programmed autophagy in the drosophila fat body is induced by ecdysone through regulation of the PI3K pathway. Dev Cell. 2004; 7:179–92. 10.1016/j.devcel.2004.07.005. 15296715

[64] Jiang C, Baehrecke EH, Thummel CS Steroid regulated programmed cell death during Drosophila metamorphosis. Development. 1997; 124:4673–83. 10.1242/dev.124.22.4673. 9409683

[65] Beckstead RB, Lam G, Thummel CS Specific transcriptional responses to juvenile hormone and ecdysone in Drosophila. Insect Biochem Mol Biol. 2007; 37:570–8. 10.1016/j.ibmb.2007.03.001. 17517334 PMC1976265

[66] Denton D, Xu T, Dayan S et al. Dpp regulates autophagy-dependent midgut removal and signals to block ecdysone production. Cell Death Differ. 2019; 26:763–78. 10.1038/s41418-018-0154-z. 29959404 PMC6460390

[67] Truman JW, Talbot WS, Fahrbach SE et al. Ecdysone receptor expression in the CNS correlates with stage-specific responses to ecdysteroids during Drosophila and Manduca development. Development. 1994; 120:219–34. 10.1242/dev.120.1.219. 8119129

[68] Stoiber M, Celniker S, Cherbas L et al. Diverse hormone response networks in 41 independent drosophila cell lines. G3 (Bethesda). 2016; 6:683–94. 10.1534/g3.115.023366. 26772746 PMC4777130

[69] Mazina MY, Kovalenko EV, Evdokimova AA et al. RNA polymerase II “pause” prepares promoters for upcoming transcription during drosophila development. Int J Mol Sci. 2022; 23:10662 10.3390/ijms231810662. 36142573 PMC9503990

[70] Kreher J, Kovač K, Bouazoune K et al. EcR recruits dMi-2 and increases efficiency of dMi-2-mediated remodelling to constrain transcription of hormone-regulated genes. Nat Commun. 2017; 8:14806 10.1038/ncomms14806. 28378812 PMC5382322

[71] Zraly CB, Middleton FA, Dingwall AK Hormone-response genes are direct in vivo regulatory targets of brahma (SWI/SNF) complex function. J Biol Chem. 2006; 281:35305–15. 10.1074/jbc.M607806200. 16990270

[72] Carrera I, Zavadil J, Treisman JE Two subunits specific to the PBAP chromatin remodeling complex have distinct and redundant functions during drosophila development. Mol Cell Biol. 2008; 28:5238–50. 10.1128/MCB.00747-08. 18573871 PMC2519717

[73] Tilly BC, Chalkley GE, van der Knaap JA et al. In vivo analysis reveals that ATP-hydrolysis couples remodeling to SWI/SNF release from chromatin. eLife. 2021; 10:e69424 10.7554/eLife.69424. 34313222 PMC8352592

[74] Bodai L, Zsindely N, Gáspár R et al. Ecdysone induced gene expression is associated with acetylation of histone H3 lysine 23 in Drosophila melanogaster. PLoS One. 2012; 7:e40565 10.1371/journal.pone.0040565. 22808194 PMC3393682

[75] Kirilly D, Wong JJL, Lim EKH et al. Intrinsic epigenetic factors cooperate with the steroid hormone ecdysone to govern dendrite pruning in Drosophila. Neuron. 2011; 72:86–100. 10.1016/j.neuron.2011.08.003. 21982371

[76] Koval LA, Proshkina EN, Zemskaya NV et al. *Drosophila melanogaster* lifespan is regulated by nejire gene expression in peripheral tissues and nervous system. Mol Biol. 2023; 57:848–66. 10.1134/S0026893323050060. 37752649

[77] Hsu E, Zemke NR, Berk AJ Promoter-specific changes in initiation, elongation, and homeostasis of histone H3 acetylation during CBP/p300 inhibition. eLife. 2021; 10:e63512 10.7554/eLife.63512. 33704060 PMC8009678

