# Supplementary material for: Transcriptional induction by ecdysone in Drosophila salivary glands involves an increase in chromatin accessibility and acetylation
Source: Nucleic Acids Res. 2025 Apr 15;53(7):gkaf284. doi: 10.1093/nar/gkaf284 (PMC11997763; doi:10.1093/nar/gkaf284)
Supplement: gkaf284_Supplemental_Files [file gkaf284_supplemental_files.zip › Supplementary figures.docx]

**Supplementary figures**

**Fig S1**

**Characterization of the EcR-full and TBP antibodies**

Newly generated antibodies against full-length EcR (A) and previously generated antibodies against TBP (B) precipitated proteins of the expected size (marked with asterisk) from the protein nuclear extract of S2 cells. The amount of precipitated proteins was reduced by EcR and TBP RNAi, respectively. To demonstrate that the equal amount of protein extract was taken for the precipitation from cells treated with dsRNA against GFP and targets, the western blots were stained with anti-lamin antibodies.

**Fig S2**

**E23 transporter is not accumulated into the nucleus of the cells**

E23 transporter was expressed in *Drosophila* S2 cells stable line bearing *MtnA*-FLAG-E23 cassette (to induce the transgene cassette cells were treated with CuSO_4_). Then, cells were immunostained with antibodies against FLAG epitope (shown in green and marked “FLAG-E23”). DAPI (shown in blue) was used for the visualization of the nuclei.


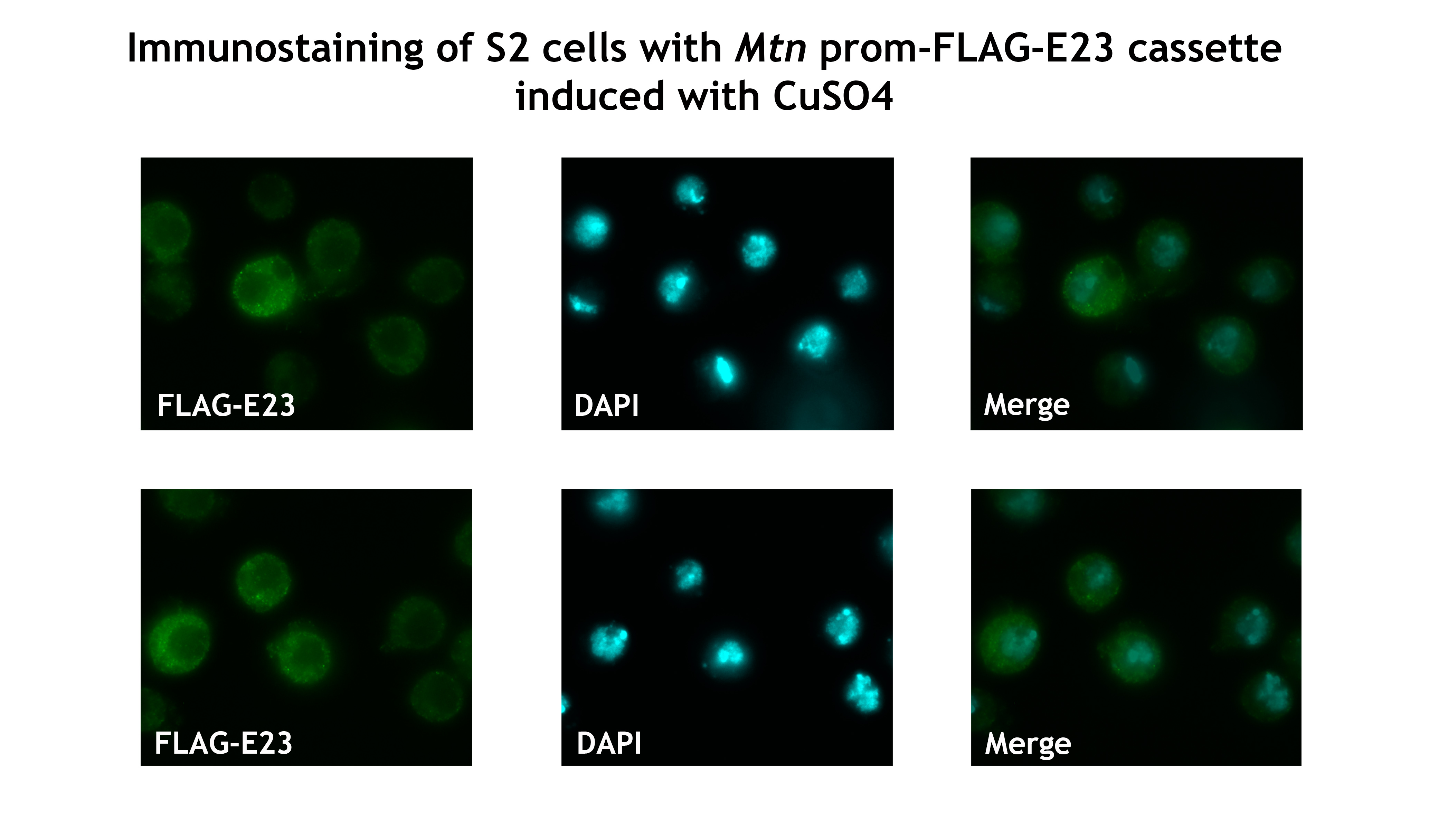


**Fig S3**

**The phenotypes of *Drosophila* tissues upon artificial expression of E23 transporter**

The tissues phenotype of *uas-e23* drosophila stock crossed with GAL4 drivers: *fkh-GAL4* (Bloomington stock #78060, expressing GAL4 in salivary glands, starting from the embryonic stage) and *tj-GAL4* (AA274 stock, a generous gift of A. Aravin, priming GAL4 expression in somatic cells of ovary). Salivary glands and ovaries were dissected in PBS/0.1% Tween, fixed with formaldehyde and stained with DAPI. The tissues were captured using the same magnification.

**
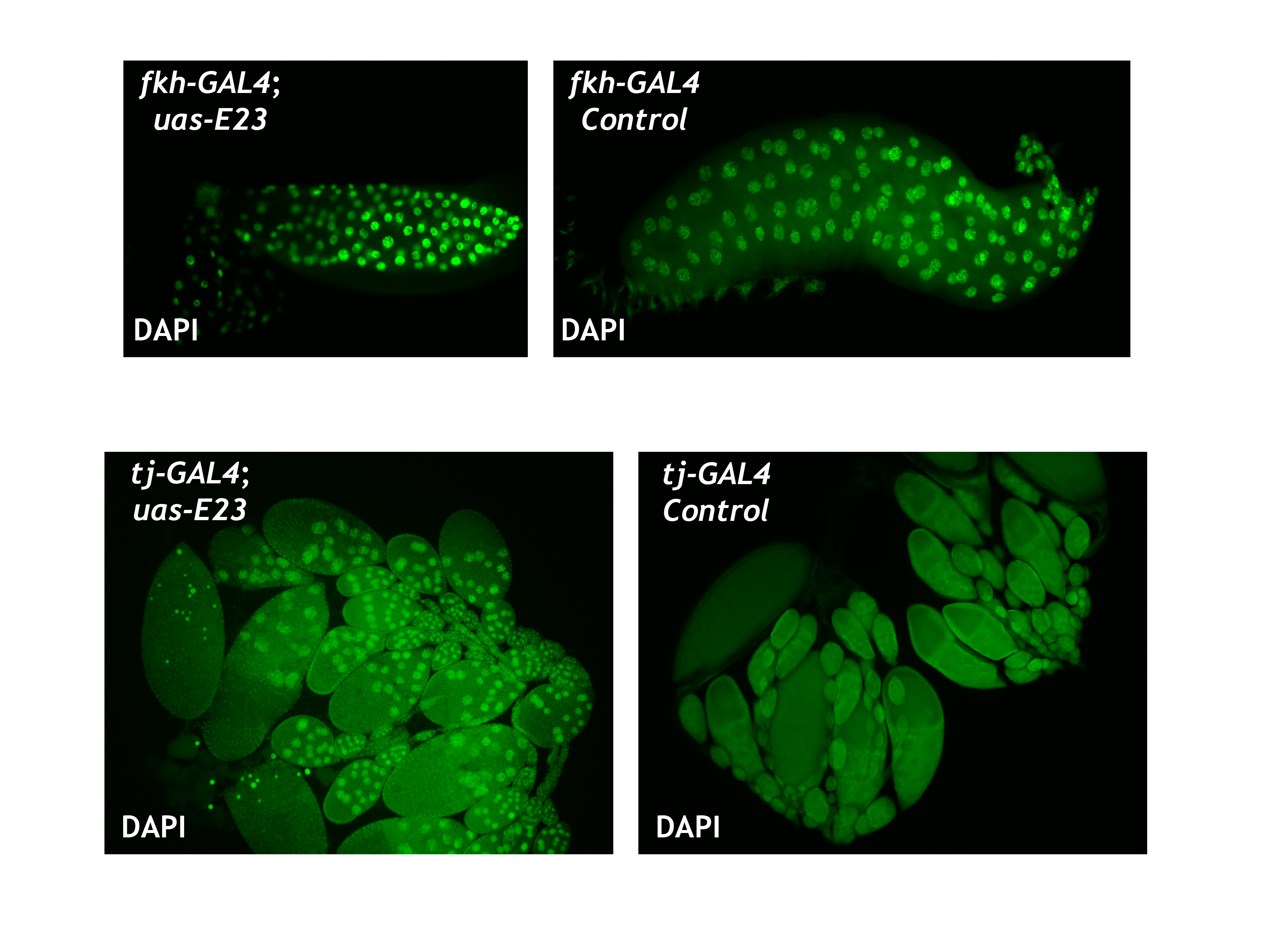
**

**Fig S4**

**GSEA analysis of 20E-activated target genes in larval salivary glands (which transcription was downregulated upon E23 expression) (N=637)**

GSEA analysis of genes for which transcriptional levels were decreased upon heat shock treatment in salivary glands of *hsp-e23* larvae (relative to control, non-heat shock conditions). Figure shows GSEA groups enriched in NHS (E23 down) conditions relative to HS (E23 up) conditions. For the RNA-Seq experiment larvae 20-22h before puparium formation were treated with double 1-hour heat shocks at 37°C with 1-hour rest at RT. Tissues were collected 4-6h before pupariation. RNA-Seq analysis was performed using polyA+ fraction.

**
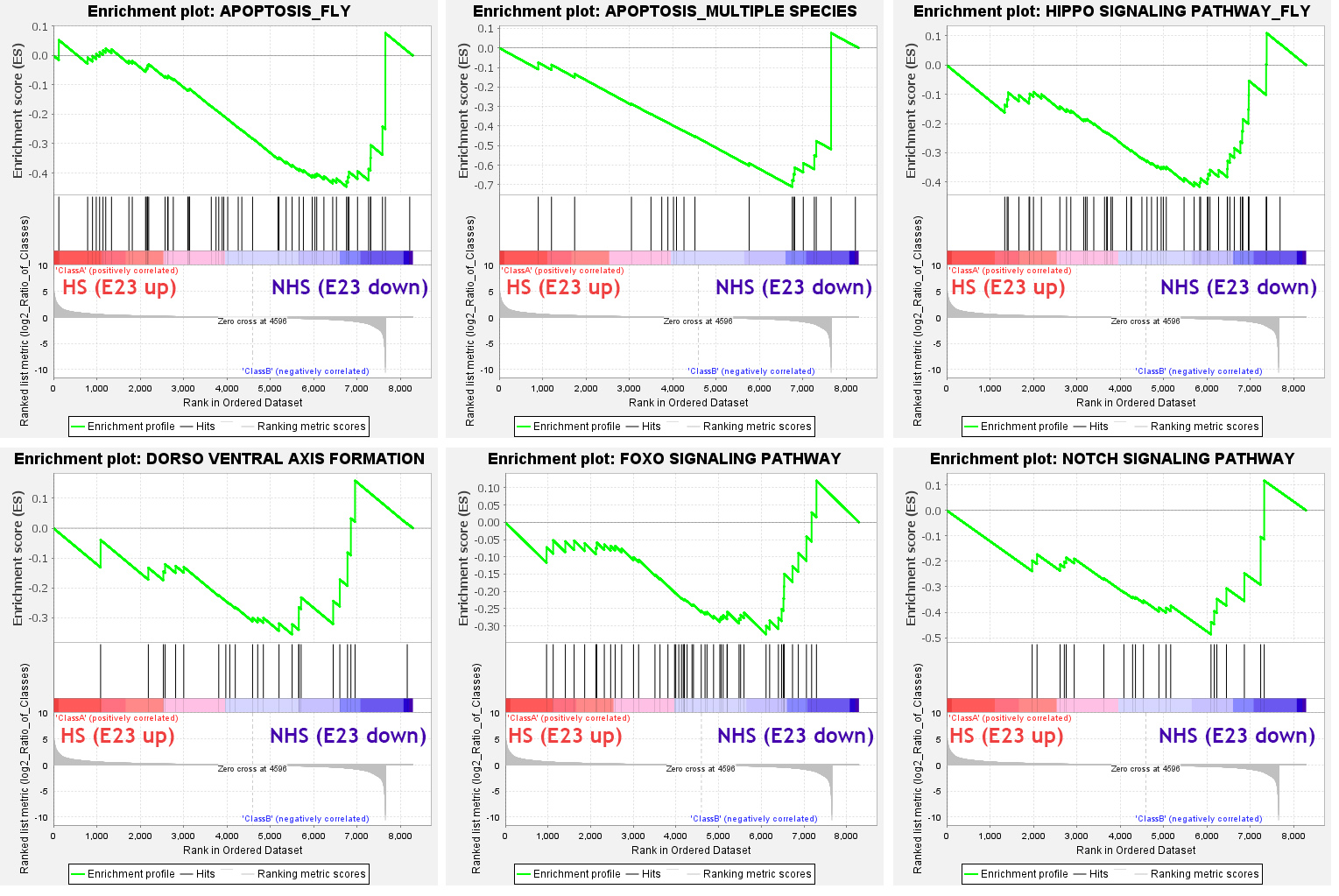
**

**Fig S5**

**GSEA analysis of 20E-suppressed target genes in larval salivary glands (which transcription was upregulated upon E23 expression) (N=598)**

GSEA analysis of genes for which transcriptional levels were increased upon heat shock treatment in salivary glands of *hsp-e23* larvae (relative to control, non-heat shock conditions). Figure shows GSEA groups enriched in HS (E23 up) conditions relative to NHS (E23 down) conditions. For the RNA-Seq experiment larvae 20-22h before puparium formation were treated with double 1-hour heat shocks at 37°C with 1-hour rest at RT. Tissues were collected 4-6h before pupariation. RNA-Seq analysis was performed using polyA+ fraction.


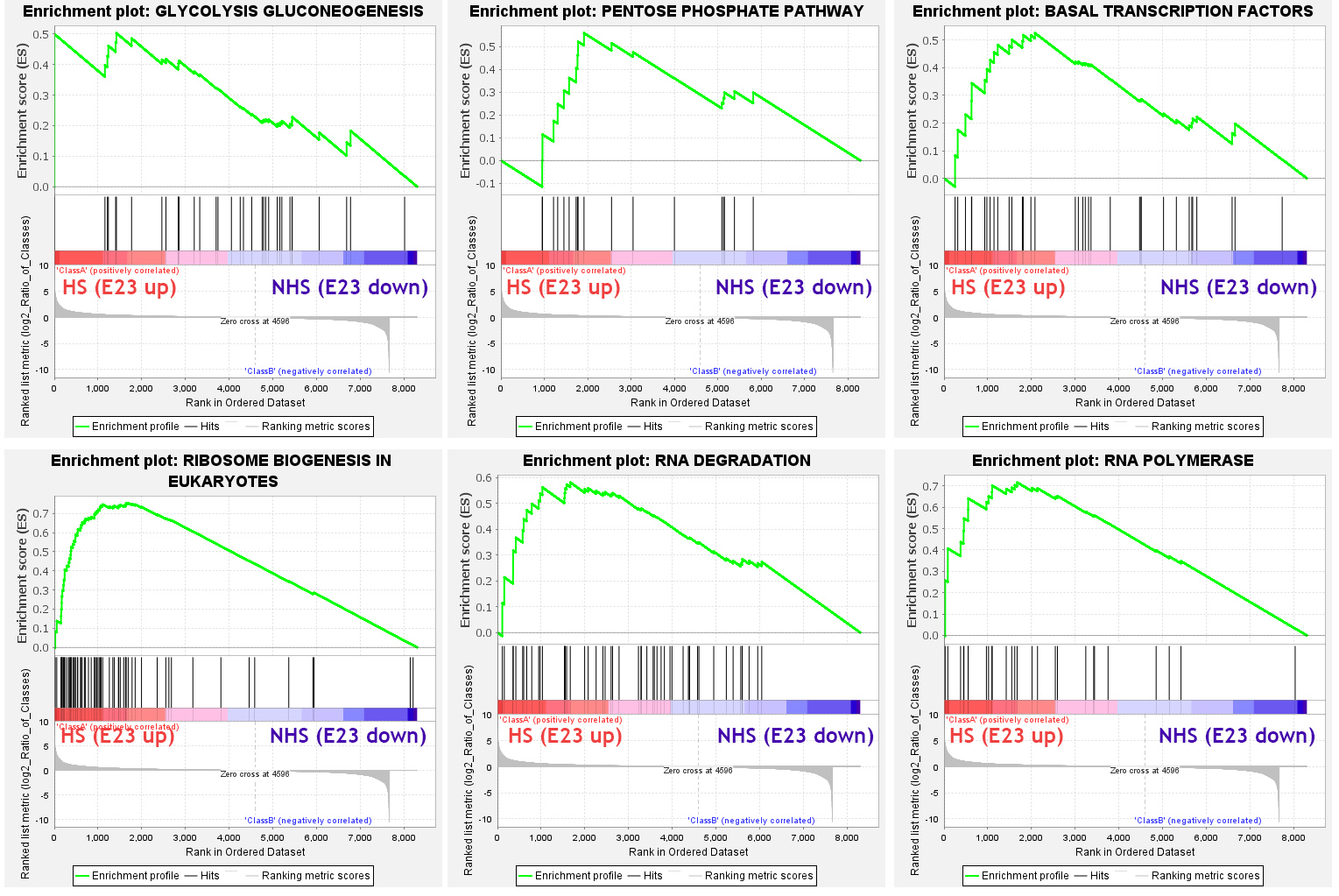


**Fig S6**

**GSEA analysis of 20E-suppressed target genes in larval brain (which transcription was upregulated upon E23 expression) (N=165)**

GSEA analysis of genes for which transcriptional levels were increased upon heat shock treatment in brain of *hsp-e23* larvae (relative to control, non-heat shock conditions). Figure shows GSEA groups enriched in HS (E23 up) conditions relative to NHS (E23 down) conditions. For the RNA-Seq experiment larvae 20-22h before puparium formation were treated with double 1-hour heat shocks at 37°C with 1-hour rest at RT. Tissues were collected 4-6h before pupariation. RNA-Seq analysis was performed using polyA+ fraction.


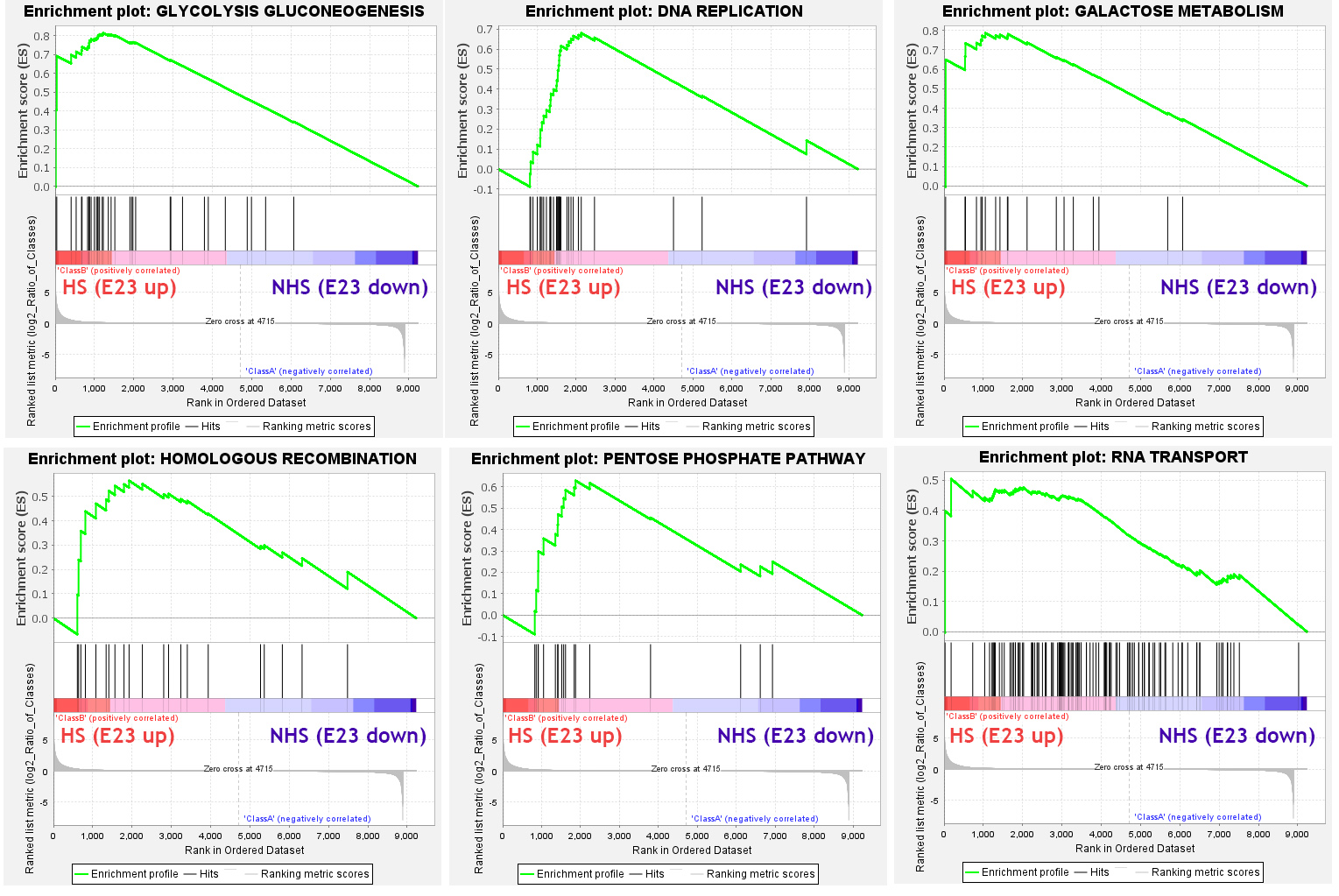


**Fig S7**

**Correlation analysis of** **ChIP-Seqs with antibodies against Rpb3, TBP and EcR performed on the salivary glands of *hsp-e23* wandering larvae**

Correlation analysis showing reproducibility between biological ChIP-seq replicates performed on a pooled set of sites representing 20E-activated (N=105) and 20E-suppressed (N=259) TSSs in the salivary glands of wandering *hsp-e23* larvae. Normalized coverage of individual replicates for ChIP-Seqs with antibodies against Rpb3, TBP, and EcR were taken for analysis (coverage was estimated as a number of reads normalized by the size of the library). Analyzed ChIP-Seqs were performed on salivary glands material from *hsp-E23* wandering larvae (untreated “NHS” or pre-treated “HS’’ with double heat shocks according to the scheme on Fig. 2A). Correlation analysis is presented as heatmaps with indicated coefficients of Pearson correlation.

**Fig S8**

**Average analysis of ChIP-Seqs with antibodies against Rpb3, TBP and EcR performed on salivary glands of *hsp-e23* wandering larvae at the control set representing all TSSs of Drosophila genome**

Average distribution of Rpb3, TBP and EcR binding estimated by ChIP-Seqs at all TSSs of *Drosophila* genome in control conditions (NHS – green line) and after the treatment of *hsp-e23* larvae 20-22h before pupariation with double 1-hour heat shock (with a 1-hour rest at RT) (HS – purple line). ChIP-Seq binding level was calculated as a ratio to Input. The X-axis represents the distance to the TSS in kbp. Average profiles were calculated as a median of binding level. The standard error is displayed on the profiles as semi-transparent area around the main line of the profiles. The fold change (FC) was calculated using normalized coverage within 500 bp around the center of the analyzed TSSs (as a ratio of NHS signal to HS signal). The results of the paired t-test analysis are provided on the graphs, where “**” means p ≤ 0.01.

**Fig S9**

**20E depletion does not lead to the recruitment of Pol II to 20E-suppressed TSSs in the salivary glands**

Quantitative analysis of the Rpb3, TBP and EcR binding level using DiffBind package at the 20E-suppressed TSSs (N=259) in *Drosophila* salivary glands **(presented at the left top panel)**. Protein binding level was estimated using ChIP-Seq analysis on the material of salivary glands of *hsp-e23* larvae in control conditions (NHS) and pre-treated with double heat shock (HS). Graphs represent a number of TSSs bearing peaks of corresponding proteins and showing statistically significant changes upon the 20E depletion (achieved by E23 expression) (FC|≥1|; p ≤ 0.05). **At the right top panel** presented the average analysis of Rpb3, TBP and EcR binding to 259 20E-suppressed TSSs (N=259) in salivary glands upon the 20E depletion. ChIP-Seq binding level was calculated as a ratio to Input. The X-axis represents the distance to the TSS in kbp. Average profiles were calculated as a median of binding level. The fold change (FC) was calculated using normalized coverage within 500 bp around the center of the analyzed TSSs (as a ratio of NHS signal to HS signal). The results of the paired t-test analysis are provided on the graphs, where “**” means p ≤ 0.01. **Bottom panel** shows the profiles of Rpb3, TBP and EcR binding as well as RNA-Seq signal in an example 20E-suppressed gene locus (*Drat* locus) in salivary glands of *hsp-e23* larvae in control conditions (NHS) and pre-treated with double heat shock (HS).





**Fig S10**

**Comparative analysis of promoter-associated motifs occurrence in 20E-activated (N=164) and 20E-suppressed (N=259) TSSs in salivary glands of *hsp-e23* wandering larvae (down or upregulated upon E23 expression respectively)**

(A) The presence of promoter-associated motifs was inferred from a recent whole genome analysis of *Drosophila* TSSs (Adato et al 2024). The presence of GAGA, TATA-box, Drosophila Inr (dInr) and pause button (PB) was determined in 20E-activated (N=164), 20E-suppressed (N=259) TSSs and compared to the occurrence of the motifs in all TSSs of the genome (All). Promoter-associated motif occurrence was presented as a percentage. To analyze the enrichment of motifs in one group compared to another we used the Fisher's Exact Test (the “*” on graphs means p ≤ 0.01). (B) The average analysis of TBP binding to 20E-activated (N=164), 20E-suppressed (N=259) TSSs and all TSSs of the genome (All) in salivary glands of *hsp-e23* wandering larvae in normal conditions (without the Heat shock). ChIP-Seq binding level was calculated as a ratio to Input. The X-axis represents the distance to the TSS in kbp. Average profiles were calculated as a median of binding level.


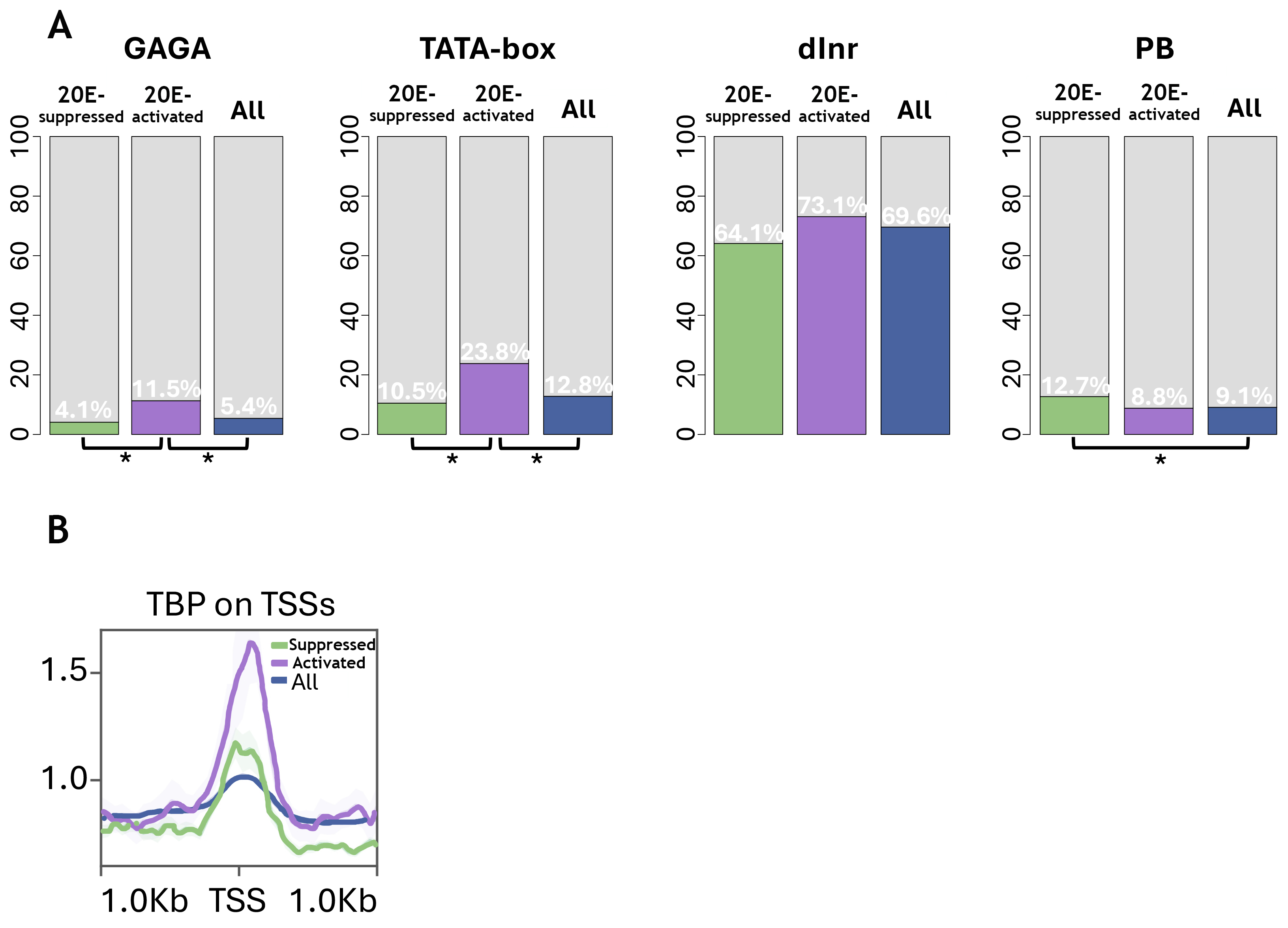


**Fig S11**

**Correlation analysis of ChIP-Seqs with antibodies against Rpb3, TBP and EcR performed on brain and salivary glands of *hsp-e23* wandering larvae**

Correlation analysis showing reproducibility between biological ChIP-seq replicates performed on a pooled set of sites representing 20E-activated (N=105) and 20E-suppressed (N=259) TSSs in the salivary glands and brain of wandering *hsp-e23* larvae. Normalized coverage of individual replicates for ChIP-Seqs with antibodies against Rpb3, TBP, and EcR were taken for analysis (coverage was estimated as a number of reads normalized by the size of the library). Analyzed ChIP-Seqs were performed on salivary glands and brain material from *hsp-E23* wandering larvae in control conditions (without heat-shock treatment). Correlation analysis is presented as heatmaps with indicated coefficients of Pearson correlation.

**Fig S12**

**Average analysis of ChIP-Seqs with antibodies against Rpb3, TBP and EcR performed on salivary glands and brain of wandering larvae at the control set representing all TSSs of *Drosophila* genome**

Average distribution of Rpb3, TBP and EcR binding estimated by ChIP-Seqs at all TSSs of *Drosophila* genome in control untreated conditions (salivary glands SG – green line, and brain BR – orange line). ChIP-Seq binding level was calculated as a ratio to Input. The X-axis represents the distance to the TSS in kbp. Average profiles were calculated as a median of binding level. The standard error is displayed on the profiles as semi-transparent area around the main line of the profiles. The fold change (FC) was calculated using normalized coverage within 500 bp around the center of the analyzed TSSs (as a ratio of SG signal to BR signal). The results of the paired t-test analysis are provided on the graphs, where “**” means p ≤ 0.01.


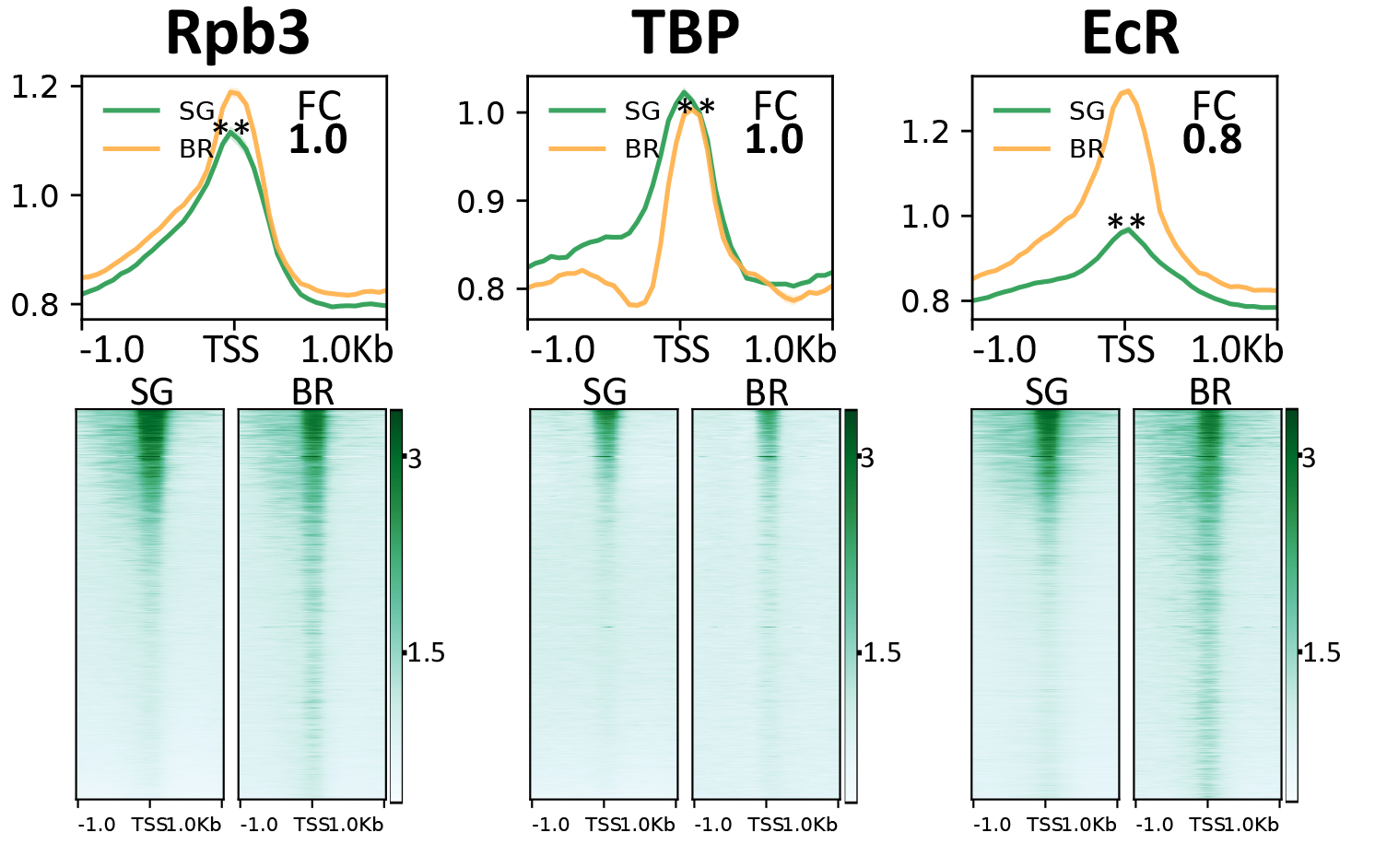


**Fig S13**

**20E-suppressed TSSs in the salivary glands bear peaks of Rpb3, TBP and EcR in brain**

Peaks distribution analysis of the Rpb3, TBP and EcR ChIP-Seqs at the 20E-suppressed TSSs (N=259) in salivary glands and brain **(presented at the left top panel)**. Protein binding level was estimated using ChIP-Seq analysis on the material of salivary glands (SG) and brain (BR) of *hsp-e23* larvae in control conditions. Graphs represent a number of TSSs bearing peaks of corresponding proteins. **At the right top panel** presented the average analysis of Rpb3, TBP and EcR binding to 259 20E-suppressed TSSs (N=259) in salivary glands and brain in control untreated conditions. ChIP-Seq binding level was calculated as a ratio to Input. The X-axis represents the distance to the TSS in kbp. Average profiles were calculated as a median of binding level. The fold change (FC) was calculated using normalized coverage within 500 bp around the center of the analyzed TSSs (as a ratio of SG signal to BR signal). The results of the paired t-test analysis are provided on the graphs, where “**” means p ≤ 0.01. **Bottom panel** shows the profiles of Rpb3, TBP and EcR binding as well as RNA-Seq signal in an example 20E-suppressed gene locus (*Drat* locus) in salivary glands and brain of *hsp-e23* larvae in control untreated conditions.

**

**

**Fig S14**

**Ecdysone-sensitive elements (ESEs) in 20E-activated primary loci in salivary glands were divided into ‘active’ (N=322) and ‘poised’ (N=504) groups according to their level of H3K27Ac**

EcR and CBP/Nejire co-bound site in 20E-activated primary target loci (+- 5 kb) – which we named ecdysone sensitive elements ESEs (N=826) – were divided based on the level of H3K27Ac modification inherent to active enhancers (using hierarchical clustering). **The left panel** shows heatmaps representing the level of H3K27Ac modification in salivary glands under untreated control conditions (in two clusters of ESEs – ‘active’ (N=322) and ‘poised’ (504)). **The right panel** demonstrates the average level of H3K27Ac in the selected groups under control (green) and 20E-depleted conditions (upon the E23 expression) (purple). The analysis of H3K27Ac level in groups were performed using normalized counts of H3K27Ac signal within ESEs +-1kb region. The results of the unpaired t-test analysis are provided on the graphs, where “**” means p ≤ 0.01.


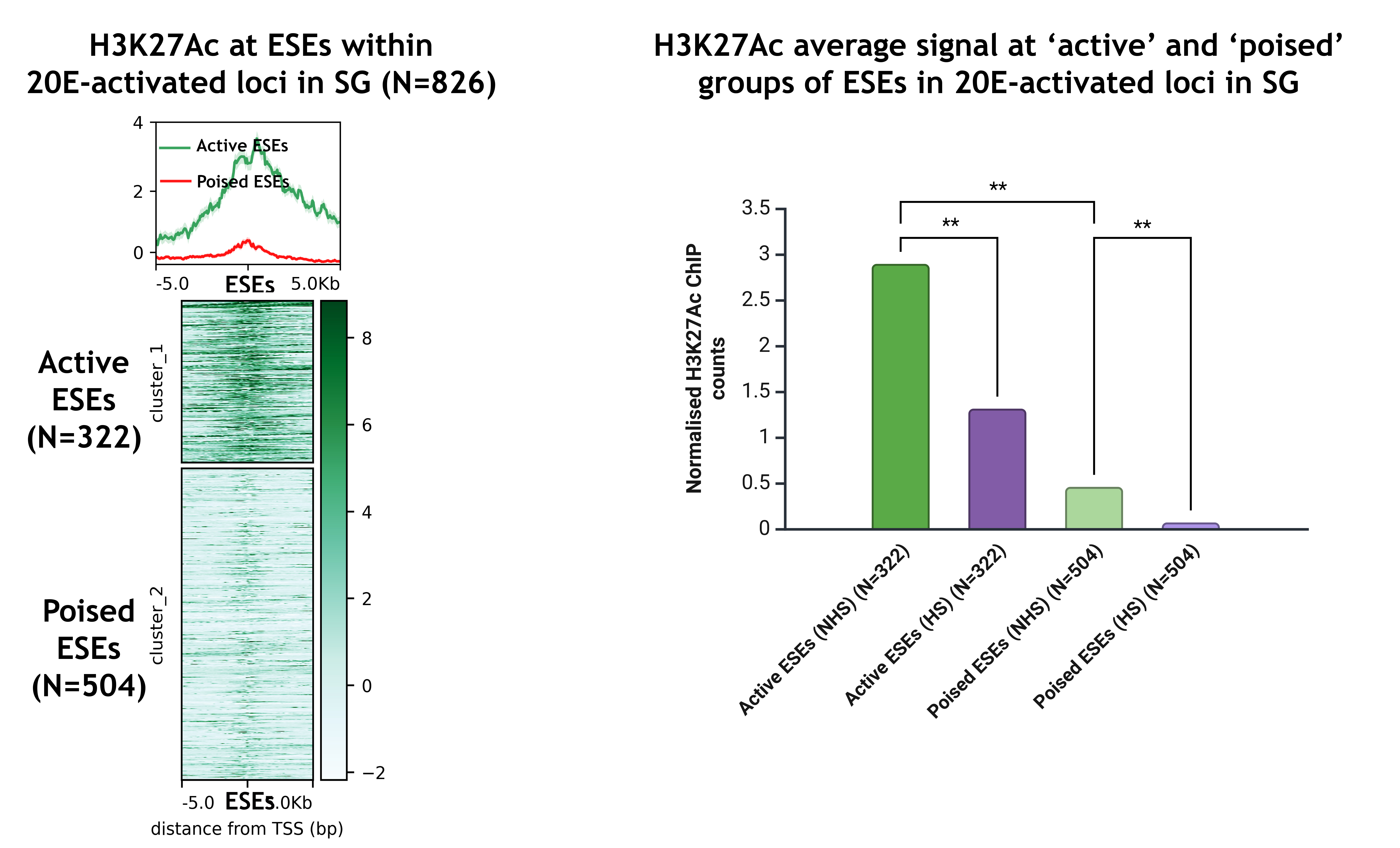


**Fig S15**

**Correlation analysis of ChIP-Seqs with antibodies against EcR, CBP/Nejire and H3K27Ac and FAIRE-Seq performed on salivary glands of *hsp-e23* wandering larvae**

Correlation analysis showing reproducibility between biological ChIP-seq replicates performed on sites representing ecdysone-sensitive elements (ESEs) located at the 20E-activated targets in the salivary glands (N=826). The correlation analysis was performed using normalized coverage of individual replicates for ChIP-Seqs with antibodies against EcR, CBP/Nejire and H3K27Ac and FAIRE-Seqs (coverage was estimated as a number of reads normalized by the size of the library) within 500 bp around the center of the analyzed ESEs. Analyzed ChIP-Seqs were performed on salivary glands material from *hsp-E23* wandering larvae (untreated “NHS” or pre-treated “HS’’ with double heat shocks according to the scheme on Fig. 2A). Correlation analysis is presented as heatmaps with indicated coefficients of Pearson correlation.

**Fig S16**

**Average analysis of ChIP-Seqs with antibodies against EcR, CBP/Nejire and H3K27Ac and FAIRE-Seq performed on salivary glands of *hsp-e23* wandering larvae at the control set representing all TSSs of *Drosophila* genome**

Average distribution of EcR, CBP/Nejire and H3K27Ac estimated by ChIP-Seq and FAIRE-Seq at all TSSs of *Drosophila* genome in control conditions (NHS – green line) and after the treatment of *hsp-e23* larvae 20-22h before pupariation with double 1-hour heat shock (with a 1-hour rest at RT) (HS – purple line). ChIP-Seq binding level was calculated as a ratio to Input. For the FAIRE-Seq and H3K27Ac the Input was subtracted. The X-axis represents the distance to the TSS in kbp. Average profiles were calculated as a median of binding level. The standard error is displayed on the profiles as semi-transparent area around the main line of the profiles. The fold change (FC) was calculated using normalized coverage within 500 bp around the center of the analyzed ESEs for EcR, CBP, FAIRE and within 1000 bp around the center of the analyzed ESEs for H3K27Ac (as a ratio of NHS signal to HS signal). The results of the paired t-test analysis are provided on the graphs, where “**” means p ≤ 0.01.

**Fig S17**

**20E depletion leads to an increase in H3K27Ac level at the ESEs located in 20E-suppressed target loci in the salivary glands**

**(A)** Distribution of the EcR and CBP/Nejire binding in 20E-suppressed primary target loci (N=234) in the salivary glands estimated both in control untreated conditions and after the Heat shock. ESEs representing EcR and CBP/Nejire co-bound sites both in NHS and HS conditions were taken for further analysis (N=637). **(B)** Quantitative analysis of the EcR and CBP/Nejire binding level using DiffBind package at the 20E-suppressed target loci in *Drosophila* salivary glands. Protein binding level was estimated using ChIP-Seq analysis on the material of salivary glands of *hsp-e23* larvae in control conditions (NHS) and pre-treated with double heat shock (HS). Graphs represent a number of ESEs bearing peaks of corresponding proteins and showing statistically significant changes upon the 20E depletion (achieved by E23 expression) (FC|≥1|; p ≤ 0.05). **(C)** An average analysis of EcR, CBP/Nejire and H3K27Ac binding and FAIRE-Seq level at the ESEs of 20E-suppressed target loci in salivary glands upon the 20E depletion. ChIP-Seq binding level was calculated as a ratio to Input. For the FAIRE-Seq and H3K27Ac the Input was subtracted. The X-axis represents the distance to the ESEs in kbp. Average profiles were calculated as a median of binding level. The fold change (FC) was calculated using normalized coverage within 500 bp around the center of the analyzed ESEs for EcR, CBP, FAIRE and within 1000 bp around the center of the analyzed ESEs for H3K27Ac (as a ratio of NHS signal to HS signal). The results of the paired t-test analysis are provided on the graphs, where “**” means p ≤ 0.01.


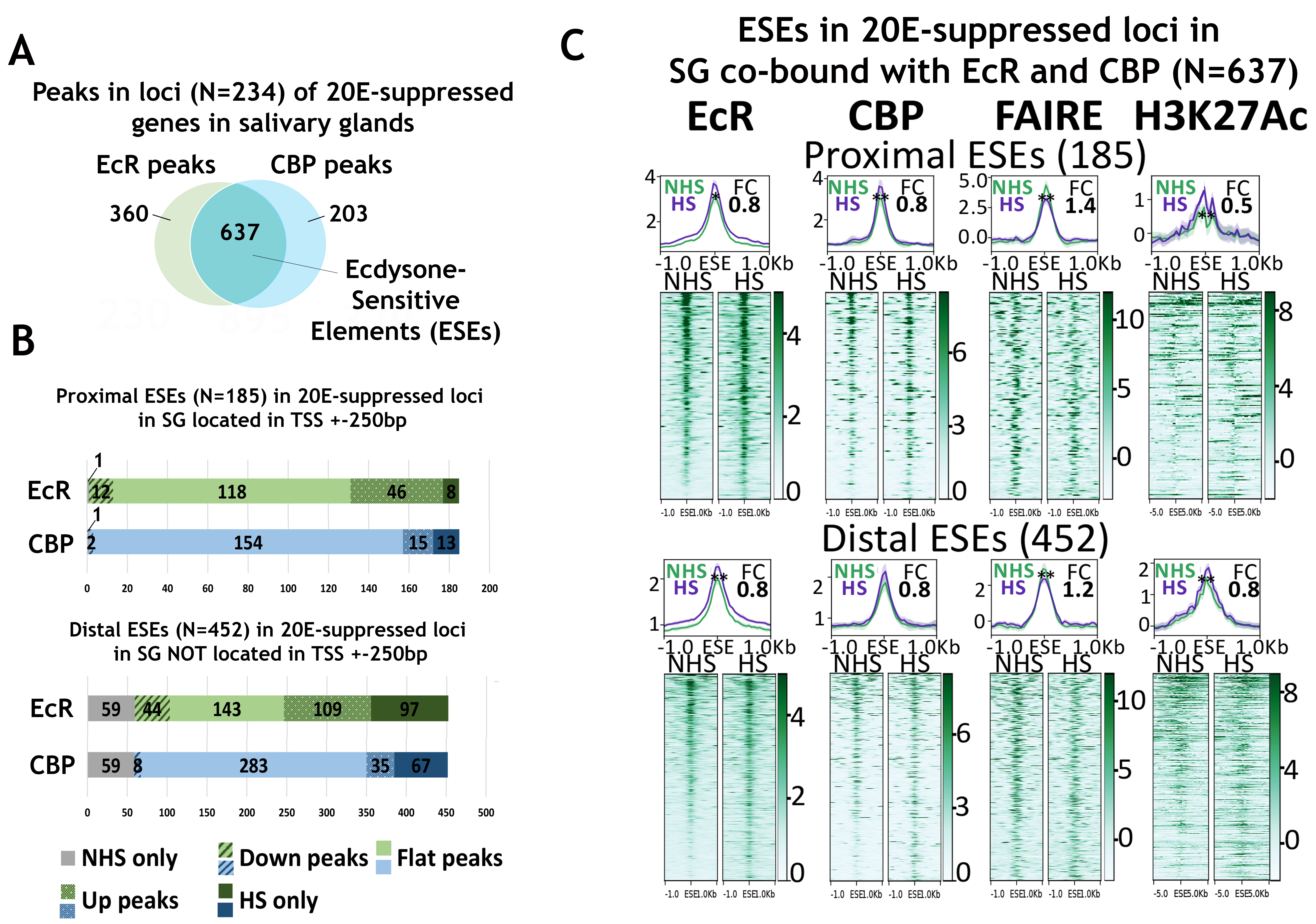


**Fig S18**

**Individual loci representing the examples of 20E-activated (*eip74ef*) and 20E-suppressed (*drat*) target genes in salivary glands**

Protein binding level was estimated by ChIP-Seqs using antibodies against EcR, CBP/Nejire and H3K27Ac. The chromatin accessibility was assessed using FAIRE-Seq analysis. Protein binding level was estimated on the material of salivary glands of *hsp-e23* wandering larvae in control conditions (NHS) and pre-treated with double heat shock (HS).


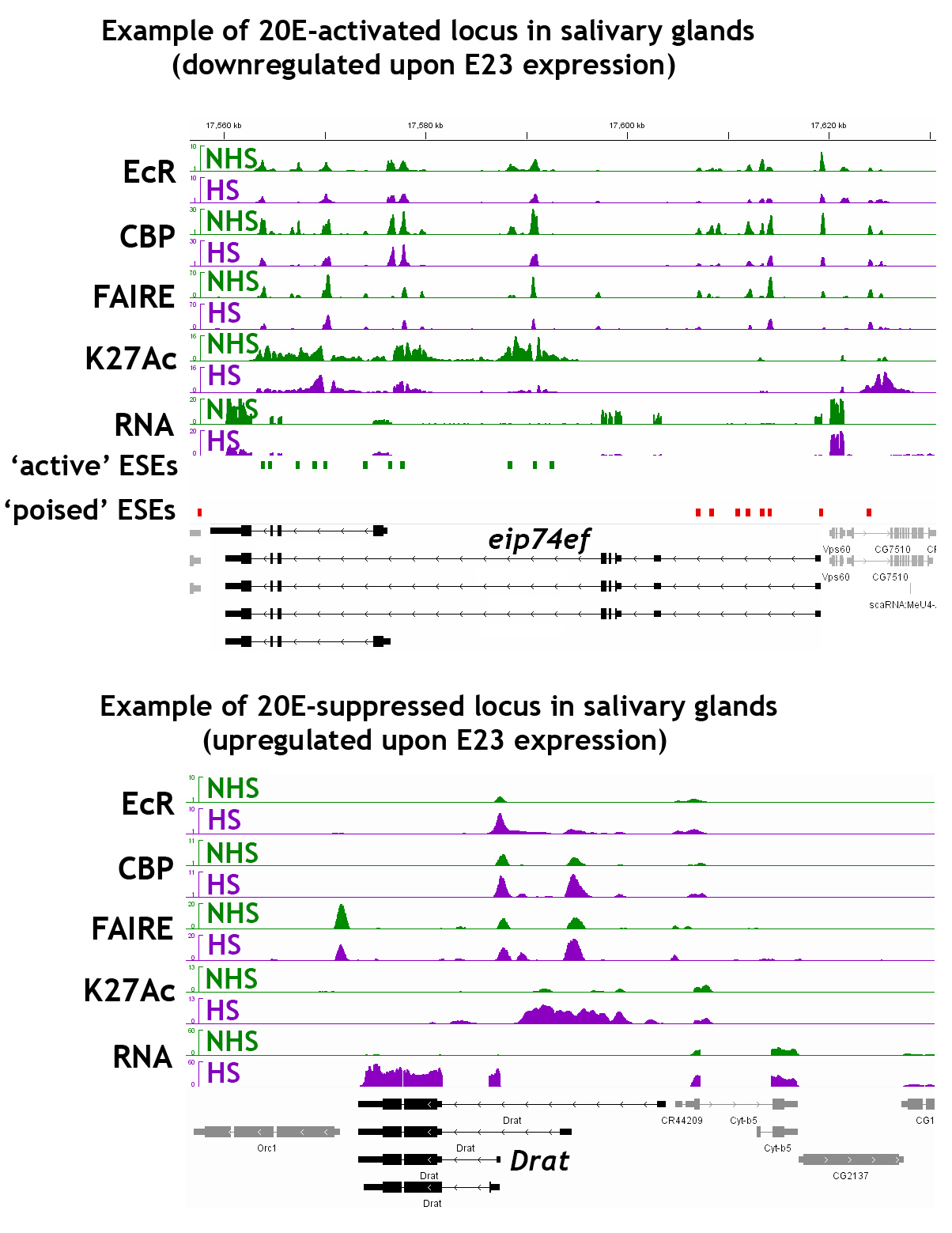


**Fig S19**

**Correlation analysis of ChIP-Seqs with antibodies against EcR, CBP/Nejire, H3K27Ac and FAIRE-Seq performed on brain and salivary glands of *hsp-e23* wandering larvae**

Correlation analysis showing reproducibility between biological ChIP-seq replicates performed on sites representing ecdysone-sensitive elements (ESEs) located in the 20E-activated targets in the salivary glands (N=826). The correlation analysis was performed using normalized coverage of individual replicates for ChIP-Seqs with antibodies against EcR, CBP/Nejire and H3K27Ac and FAIRE-Seqs (coverage was estimated as a number of reads normalized by the size of the library) within 500 bp around the center of the analyzed ESEs. Analyzed ChIP-Seqs were performed on salivary glands and brain material from *hsp-E23* wandering larvae in control conditions (without heat-shock treatment). Correlation analysis is presented as heatmaps with indicated coefficients of Pearson correlation. **Bottom panel** of the figure represent the example *eip74ef* locus of 20E-activated targets in salivary glands. Protein binding level was estimated on the material of brain and salivary glands of *hsp-e23* wandering larvae in control untreated conditions.

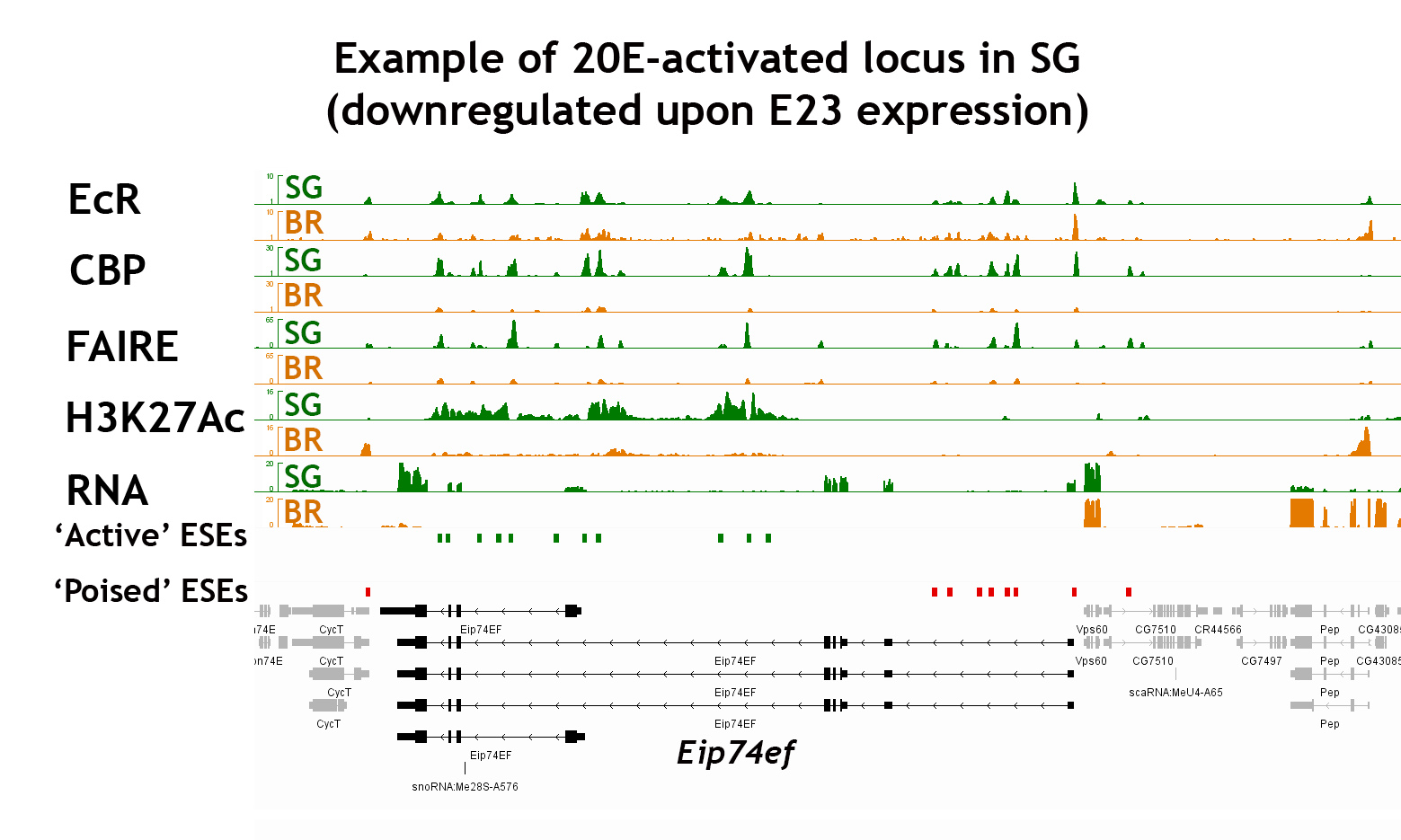


**Fig S20**

**Average analysis of ChIP-Seqs with antibodies against EcR, CBP/Nejire, H3K27Ac and FAIRE-Seq performed on salivary glands and brain of wandering larvae at the control set representing all TSSs of Drosophila genome**

Average distribution of EcR, CBP/Nejire, H3K27Ac binding estimated by ChIP-Seqs and FAIRE-Seqs at all TSSs of Drosophila genome in control untreated conditions (salivary glands SG – green line, and brain BR – orange line). ChIP-Seq binding level was calculated as a ratio to Input. For the FAIRE-Seq the Input was substracted. The X-axis represents the distance to the TSS in kbp. Average profiles were calculated as a median of binding level. The standard error is displayed on the profiles as semi-transparent area around the main line of the profiles. The fold change (FC) was calculated using normalized coverage within 500 bp around the center of the analyzed ESEs for EcR, CBP, FAIRE and within 1000 bp around the center of the analyzed ESEs for H3K27Ac (as a ratio of SG signal to BR signal). The results of the paired t-test analysis are provided on the graphs, where “**” means p ≤ 0.01.

**Fig S21**

**ESEs at 20E-suppressed target loci in the salivary glands are tissue-specific**

**At the left panel** presented the average analysis of EcR, CBP/Nejire and H3K27Ac binding and FAIRE-Seq to ESEs at 20E-suppressed target loci. ChIP-Seqs and FAIRE-Seqa analysis were performed on the material of salivary glands (SG) and brain (BR) of *hsp-e23* larvae in control conditions. ChIP-Seq binding level was calculated as a ratio to Input. For the FAIRE-Seq and H3K27Ac the input was subtracted. The X-axis represents the distance to the ESEs in kbp. Average profiles were calculated as a median of binding level. The fold change (FC) was calculated using normalized coverage within 500 bp around the center of the analyzed ESEs for EcR, CBP, FAIRE and within 1000 bp around the center of the analyzed ESEs for H3K27Ac (as a ratio of NHS signal to HS signal). The results of the paired t-test analysis are provided on the graphs, where “**” means p ≤ 0.01. **Right panel** shows the profiles of EcR, CBP/Nejire and H3K27Ac binding as well as FAIRE-Seq signal in an example 20E-suppressed gene locus (*Drat* locus) in salivary glands and brain of *hsp-e23* larvae in control untreated conditions.


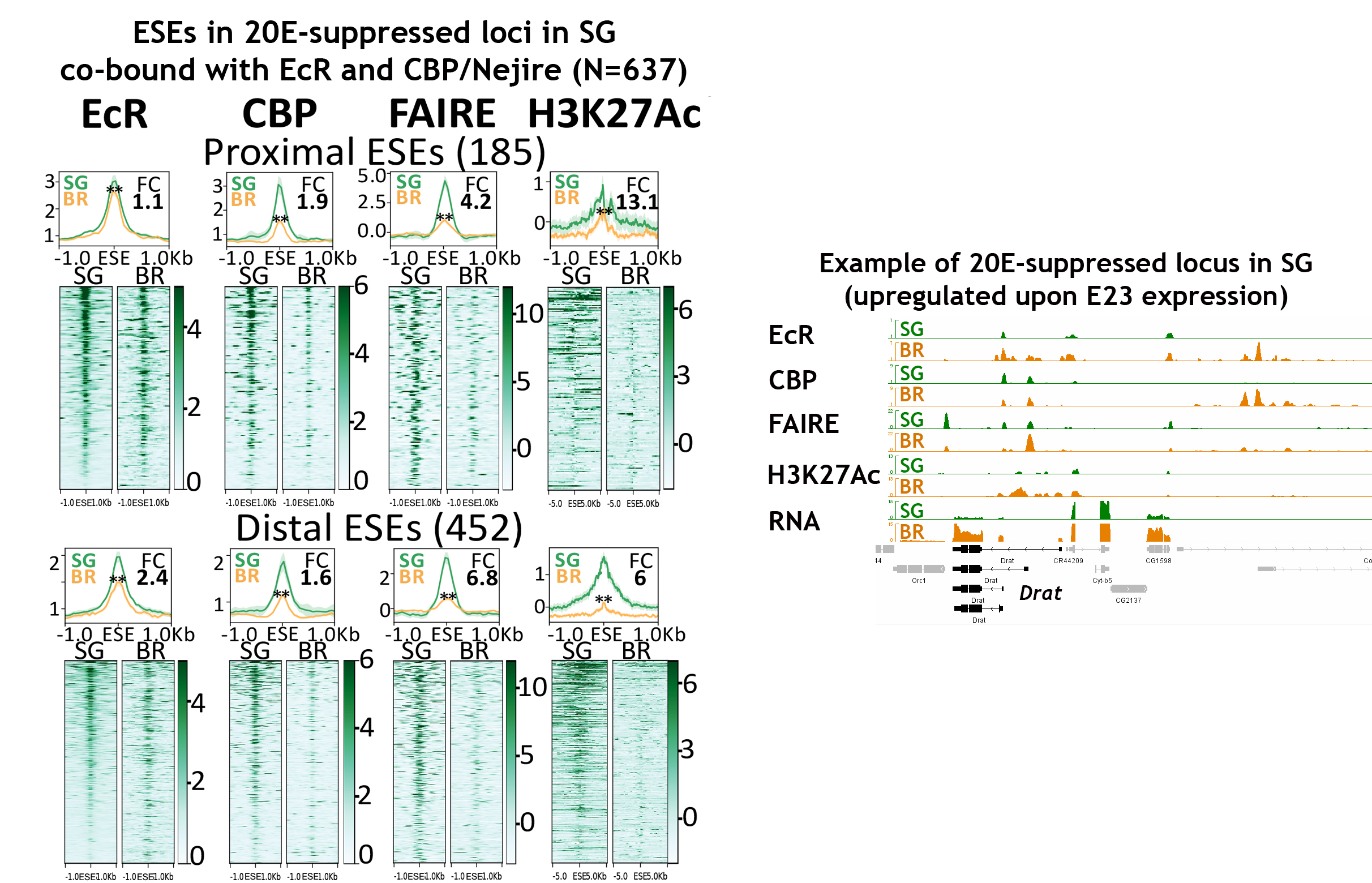


**Fig S22**

**Transcription of 20E-suppressed targets in salivary glands is maintained in brain tissue through the enhancers different from ESEs in the salivary glands**

An average analysis of EcR, CBP/Nejire and H3K27Ac binding and FAIRE-Seq to CBP/Nejire sites in brain at 20E-suppressed target loci located outside the salivary-glands-specific ESEs (N=90). ChIP-Seqs and FAIRE-Seq analysis were performed on the material of salivary glands (SG) and brain (BR) of *hsp-e23* larvae in control conditions. ChIP-Seq binding level was calculated as a ratio to Input. For the FAIRE-Seq and H3K27Ac the input was subtracted. The X-axis represents the distance to the CBP peaks in kbp. Average profiles were calculated as a median of binding level. The fold change (FC) was calculated using normalized coverage within 500 bp around the center of the analyzed CBP peaks for EcR, CBP, FAIRE and within 1000 bp around the center of the analyzed CBP peaks for H3K27Ac (as a ratio of SG signal to BR signal). The results of the paired t-test analysis are provided on the graphs, where “**” means p ≤ 0.01.

**Fig S23**

**Transcriptional level of genes encoding components of Dpp signaling pathway in salivary glands and brain of *hsp-e23* wandering larvae**

RNA-Seq (polyA+) data representing transcriptional level of loci coding components of Dpp signaling pathway in brain and salivary glands tissues of *hsp-e23* and Oregon wandering larvae under control untreated conditions. Transcriptional level is presented as a number of reads normalized to genome size.

**
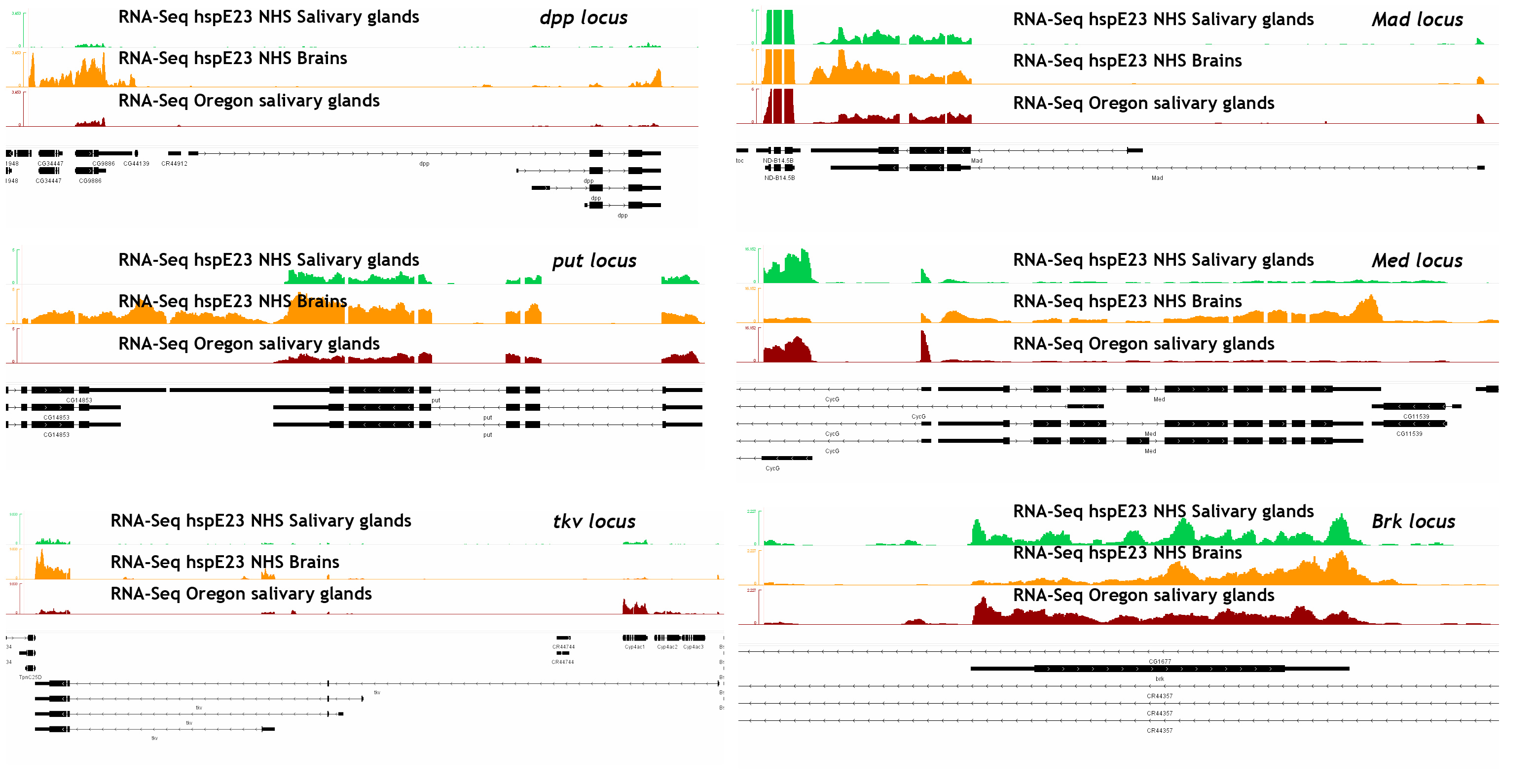
**

**Fig S24**

**Many 20E-activated targets in salivary glands of wandering larvae (defined in the current study) are present in previous screenings for targets of 20E or EcR**

Venn diagrams representing intersection of 20E-activated target genes identified in this study in salivary glands of wandering larvae (green) with targets identified by RNAi interference of EcR in salivary glands of wandering larvae (from Uyehara et al 2022) or by treatment of various cultured cell lines with 20E (from Stoiber et al 2016) (shown in blue).
