# Supplementary material for: Transcriptional induction by ecdysone in Drosophila salivary glands involves an increase in chromatin accessibility and acetylation
Source: Nucleic Acids Res. 2025 Apr 15;53(7):gkaf284. doi: 10.1093/nar/gkaf284 (PMC11997763; doi:10.1093/nar/gkaf284)
Supplement: gkaf284_Supplemental_Files [file gkaf284_supplemental_files.zip › Supplementary methods.docx]

**Supplementary methods**

**The dissection of the wandering larvae tissues**

All flies were maintained at 25°C on the standard yeast medium. For the RNA-Seq and ChIP-Seq experiments, *hsp-e23* larvae of L3 stage 20-22h before puparium formation were subjected to double 1-hour heat shocks at 37°C with a 1-hour rest period between at RT. For the dissection, wandering larvae were collected approximately 4-6 hours before the puparium formation. Dissections were performed manually in PBS/0.1% NP-40. Tissues targeted for RNA-Seqs were collected directly into TRI-reagent. Dissected salivary glands and brain aimed for ChIP-Seqs and FAIRE-Seqs were accumulated in tubes containing PBS/0.1% NP-40 and placed on ice. Dissected tissues were stored on ice not more than 1 hour. After the dissection, tissues were warmed up to room temperature and fixated by addition of formaldehyde up to 1% for 10 min. The fixation was stopped by addition of 1/20V 2.5M Gly to the reaction for 5 min. After the fixation, the Sonication buffer (ChIP lysis buffer) containing 0.1% SDS and PIC (Roche) was added to the tissues and they were stored at -70°C. The sonication step was performed after the thawing. Tissues aimed at the FAIRE-Seq analysis were subjected to -70°C and stored without addition of a buffer, as a pellet.

For each ChIP-Seq and RNA-Seq sample (single biological replicate) we used 30 salivary glands or brain of wandering larvae (for the FAIRE-Seq we used 15). Salivary glands and brain were collected in parallel from the same larvae and used for the parallel experiments.

**RNA-Seq, ChIP-Seq and FAIRE-Seq data acquiring and analysis**

All the data for RNA-Seq, ChIP-Seq and FAIRE-Seq were obtained in two independent biological replicates for each condition. Raw and processed files were deposited into the Gene Expression Omnibus—**GSE260939** (token for an access – **qtezmgiwnvenbwl**). All the regions analyzed in the manuscript are provided as .bed files in the Supplementary tables.

Total RNA was extracted with the TRI reagent (Ambion). PolyA comprising RNA fraction was isolated and prepared for sequencing with the NEBNext Ultra™ II Directional RNA Library Prep Kit. New generation sequencing was performed by Evrogen (evrogen.ru) with the Illumina NovaSeq6000 sequencer. Adapter sequences were removed from the raw files by Cutadapt (1). Mapping to *Drosophila dm6* genome was performed by HISATII; only reads with MAPQ score ≥ 10 were taken for further analysis (2). Differential analysis of RNA-Seqs was employed by limma (3). Lists of transcripts differentially expressed in brain and salivary glands of *hsp-e23* wandering larvae after the heat shock are provided in a Supplementary. 20E-activated target genes in salivary glands were selected as genes whose transcription were decreased upon HS and E23 expression with log fold change ≥|1.5|; adj. p≤0.05 (N=637, after transposons skipping N=630). 20E-suppressed target genes in salivary glands and brain were selected as genes whose transcription were increased upon HS and E23 expression with log fold change ≥|1.5|; adj. p≤0.05 (N=598 – for the salivary glands and N=165 – for the brain).

Among the all 20E-activated and 20E-suppressed target genes in salivary glands (downregulated and upregulated upon E23 expression, respectively). We considered ‘primary targets’ those genes with 20E-dependent transcription and whose loci (with the flanking regions ± 5 kb) had ‘top 2000’ EcR peaks (which can be directly affected by 20E). To estimate genome-wide EcR binding, we performed ChIP-Seq on the salivary glands of *hsp-e23* wandering larvae with antibodies against full-length EcR. The ChIP-Seq by antibodies against EcR full gave us 10679 peaks in salivary glands of non-heat shocked *hsp-e23* wandering larvae. ChIP-Seq peaks by EcR full antibodies very well corresponded to ChIP-Seq peaks obtained using EcR-C end antibodies obtained previously (on the same material – salivary glands of *hsp-E23* wandering larvae). The EcR-C end antibodies provided us with 8020 peaks in the salivary glands, 7043 of which overlapped with the EcR full peaks. The total number of peaks bound by EcR full in salivary glands was corresponded to a total number of peaks called previously using Cut&Run data for EcR in wing discs (4).

From all the promoters (corresponding to 279 20E-activated primary target genes and 234 20E-suppressed primary target genes in salivary glands), we selected 20E-dependent TSSs/promoters. For that we assessed the transcriptional level (estimated as RNA-Seq signal normalized to the genome) in TSSs-proximal regions (0-500 bp of TSSs) separately in biological replicates and performed paired t-test analyses. 164 unique transcriptional start sites (TSSs) exhibiting a reduced RNA-Seq signal within 500 bp downstream of TSSs (with FC ≥|1.5|; p ≤ 0.05) (these TSSs were named 20E-activated TSSs). And 259 unique transcriptional start sites (TSSs) exhibiting an increased RNA-Seq signal within 500 bp downstream of TSSs (with FC ≥|1.5|; p ≤ 0.05) (these TSSs were named 20E-suppressed TSSs).

The chromatin immunoprecipitation (ChIP) and FAIRE-seq were performed and analyzed exactly as previously described (5–7). FAIRE-Seq was performed using previously described protocol (8). ChIP-Seq and FAIRE-Seq libraries were obtained using the NEBNext UltraTM II DNA library preparation kit (New England Biolabs). Next generation sequencing was performed by Evrogen (evrogen.ru) and LLC "SEQUENTIO" with the Illumina NovaSeq6000 sequencer. The paired‐end reads in FastQ format were mapped to the *Drosophila* genome assembly dm6 using HISAT2 (2) and filtered (with minimum MAPQ quality score = 10). Deeptool2 package was used for the further analysis of the obtained data (9). BigWig files were generated using bamCoverage 3.0.2 with scores representing number of reads normalized by the size of the library (the protein binding levels were normalized to the genome content – calculated as RPGC: number of reads per bin/(total number of mapped reads * fragment length/effective genome size) (9). The final bigwig files (representing the protein binding profiles) were obtained using bigwigcompare tool by computing a ratio between the ChIP signal and the Input (all Inputs were preliminary smoothed over a 1 kb window). For the H3K27Ac ChIP-Seqs and FAIRE-Seq the Input was subtracted from the signal. For the peak calling from the ChIP-Seq data we used MACS2 (10, 11). All peaks were called using two independent biological replicates and appropriate Input files. We performed the correlation analysis to show reproducibility between biological ChIP-seqs and FAIRE-Seqs replicates (using deeptool2 package) (9). The results of the correlation analysis are provided in supplementary figures as heatmaps with indicated Pearson correlation coefficients. Correlation analyses for the promoter-associated proteins was performed on a pooled set of sites representing 20E-activated (N=105) and 20E-suppressed (N=259) TSSs in salivary glands. Correlation analyses for the enhancers-associated proteins was performed on sites representing ecdysone-sensitive elements (ESEs) located in the 20E-activated targets in the salivary glands (N=826).

To quantitively analyse the differences in protein binding in ChIP-Seqs upon the 20E depletion, we utilized DiffBind (part of the Bioconductor package, which uses the DESeq2 algorithm) (12). We considered peaks statistically significant if they met the conditions FC|≥1|; p ≤ 0.05.

Pile-up profiles for the average analysis of ChIP-Seqs were calculated as a median level of protein binding. Standard error is presented at the averaged profiles as a lighter area surrounding a graph. Comparing the average ChIP-Seqs signal at a given set of sites, we performed a statistical test to assess normalized coverage, which was calculated within 500 bp around the centre of the analysed sites (we used paired t-test). The test results were presented on average profiles, where “*” means p ≤ 0.05, and “**” means p ≤ 0.01. In addition, we provided the calculated fold change (which was calculated as the ratio between the average ChIP-Seq signals (median)).

We did not use any figures or text from the previously published manuscripts – only data deposited in free access databases. The Galaxy-P platform was used for analysis of ChIP-Seq data (13).

All processed files (both .bigwig and .bed) were obtained with coordinates of *Drosophila* genome assembly dm6.

**Analysis of promoter-associated motifs at 20E-activated and 20E-repressed TSSs in salivary glands**

Motif analysis at TSSs of 20E-dependent genes was conducted using the Eukaryotic Promoter Database (EPDnew) (14, 15). The data from CORE table (<https://www.juven-gershonlab.org/resources/core-2023/>) for 4 core promoter elements (GAGA, TATA box, dInr, and Pause button) was taken for our lists of downregulated (20E-activated, N=164) and upregulated (20E-suppressed, N=259) TSSs. As a control we used all the TSSs of *Drosophila melanogaster* genome represented in the CORE table.

The difference in occurrence frequency of each core motif was compared between TSSs groups (downregulated, upregulated, control) by the **Fisher's exact test.** The test results were presented on barplots, where “*” means p ≤ 0.05.

**Experiments with *Drosophila* S2 cells**

*Drosophila* Schneider cell line 2 (S2) cells were maintained at 25°C in ecdysone-free Schneider’s insect medium (Sigma) containing 10% FBS (HyClone). The treatment of S2 cells with 20-hydroxyecdysone (20E) (H5142, Sigma-Aldrich) was performed at a final concentration of 0.3 μM, as was described previously (5, 16). Coding region of *Drosophila melanogaster e23* gene (which is the same for all the transcripts) was cloned into the construct pRmha3 under the control of *MtnA* promoter and marked with a triple FLAG epitope. For stable transfection, *Drosophila S2* cells were transfected with the obtained expression plasmid and the pCoBlast plasmid, taken at a 20 to 1 M ratio using the Effectene Transfection Reagent (Qiagen). Polyclonal cell lines bearing stably integrated expression constructs were selected and maintained subsequently in Schneider medium supplemented with Blasticidin (SigmaAldrich) at a final concentration of 20 μg/mL

The presence of FLAG-tagged E23 protein in *Drosophila* S2 cells only after their treatment with CuSO_4_ were confirmed by western blots.

**Analysis of 20E concentration in salivary glands of wandering *hsp-e23* larvae**

For each biological replicate, we collected 250 salivary glands. Obtained tissues were homogenized in Dounce in 1 ml of methanol and 20E were extracted by incubating on a rotating device for ON at 4°C. After the incubation, we removed lipids by double extraction with 0.5 ml hexane. Remained methanol was evaporated and a sample was subjected to LC-MS analysis. Each biological replicate was analyzed in triple technical replicates.

A Waters Acquity I HPLC system coupled to a Waters TQ-S-micro triple quadruple mass spectrometer (Waters Corp, Milford, CT, USA) was used for analysis. Chromatographic separation was conducted using a Waters ACQUITY BEH C18 column 1.7µm, 100 mm x 2.1 mm (Waters, USA). Both mobile phase A (water) and phase B (acetonitrile) contained 0.1% formic acid. The linear gradients were as follows: 10% B at 0.1 min, 99% B at 2 min, 99% B at 4 min, 10% B at 4.1 min, and 10% B at 6 min. The flow rate was 0.3 mL/min, and the column temperature was set at 40℃. The injection volume was 5 µL. MS detection was achieved by transition with m/z 481.3 →445.3 with a dwell time of 50 ms. Capillary and cone voltages were 23 and 13 V, respectively. The source temperature was set at 150℃, the desolvation temperature was set at 500℃, and the source and desolvation gas flow rates were 10 L/min and 1000 L/h, respectively.

**List of Drosophila genome regions analysed in the manuscript (all regions provided in Supplementary tables as .bed files in coordinates of Dm6 genome)**

|  | Supplementary table name | Number of regions in a file |
| --- | --- | --- |
| Table S1 | 20E-activated_targets_in_SG_(downregulated_upon_E23_expression)_(637) | 637 |
| Table S2 | Differenatial analysis RNA-Seq hspE23 brains HS vs NHS |  |
| Table S3 | Differential analysis RNA-Seq hspE23 salivary glands HS vs NHS |  |
| Table S4 | 20E-suppressed_targets_in_SG  _(downregulated_upon_E23_expression)_(598) | 598 |
| Table S5 | 20E-suppressed_targets_in_Brain  _(downregulated_upon_E23_expression)_(165) | 165 |
| Table S6 | Differential analysis RNA-Seq Oregon HS vs NHS salivary glands |  |
| Table S7 | 20E-activated_primary_target_loci_in SG_with_top2000_EcR_peaks_(279)_dm6 | 279 |
| Table S8 | 20E-suppressed_primary_target_loci_in SG_with_top2000_EcR_peaks_(234)_dm6 | 234 |
| Table S9 | 20E-activated_TSSs_primaty_targets_in_SG_(164)  _(plus_minus_250bp)_FC_1.5_p_0.05_dm6 | 164 |
| Table S10 | Differential analysis of TSS-proximal transcription in 20E-activated primary targets in SG_FC 1.5 and p-value 0.05_(N164)_dm6 |  |
| Table S11 | 20E-activated_TSSs_primaty_targets_in_SG_with_Rpb3_peaks_(105)  _(plus_minus_250bp)_dm6 | 105 |
| Table S12 | Differential analysis of TSS-proximal transcription in 20E-suppressed primary targets in SG_FC 1.5 and p-value 0.05_(N259)_dm6 |  |
| Table S13 | 20E-suppressed_TSSs_primaty_targets_in_SG_(259)  _(plus_minus_250bp)_FC_1.5_p_0.05_dm6 | 259 |
| Table S14 | ESEs_(common_EcR_and_CBP_in_20E-activated_primary_targets_in_SG_(826)  _summits_dm6 | 826 |
| Table S15 | Active_ESEs_(322)_in_20E-activated_primary_targets_in_SG  _summits_dm6 | 322 |
| Table S16 | Poised_ESEs_(504)_in_20E-activated_primary_targets_in_SG  _summits_dm6 | 504 |
| Table S17 | Distal_active_ESEs_(258)_in_20E-activated_primary_targets_in_SG  _summits_dm6 | 258 |
| Table S18 | Distal_poised_ESEs_(369)_in_20E-activated_primary_targets_in_SG_summits  _dm6 | 369 |
| Table S19 | Proximal_active_ESEs_(64)_in_20E-activated_primary_targets_in_SG_summits  _dm6 | 64 |
| Table S20 | Proximal_poised_ESEs_(135)_in_20E-activated_primary_targets_in_SG  _summits_dm6 | 135 |
| Table S21 | ESEs_(common_EcR_and_CBP_in_20E-suppressed_primary_targets_in_SG_(637)  _summits_dm6 | 637 |
| Table S22 | Distal_ESEs_(NHS_and_HS)_in_20E-suppressed_primary_targets_in_SG_(452)  _summits_Dm6 | 452 |
| Table S23 | Proximal_ESEs_(NHS_and_HS)_in_20E-suppressed_primary_targets_in_SG_(185)  _summits_Dm6 | 185 |
| Table S24 | CBP_peaks_in_Brain_out_of_ESEs_but_in_20E-suppressed_loci_(90)_summits_dm6 | 90 |

1. Martin,M. (2011) Cutadapt removes adapter sequences from high-throughput sequencing reads. *EMBnet.journal*, **17**, 10–12.

2. Kim,D., Paggi,J.M., Park,C., Bennett,C. and Salzberg,S.L. (2019) Graph-based genome alignment and genotyping with HISAT2 and HISAT-genotype. *Nat Biotechnol*, **37**, 907–915.

3. Liu,R., Holik,A.Z., Su,S., Jansz,N., Chen,K., Leong,H.S., Blewitt,M.E., Asselin-Labat,M.-L., Smyth,G.K. and Ritchie,M.E. (2015) Why weight? Modelling sample and observational level variability improves power in RNA-seq analyses. *Nucleic Acids Research*, **43**, e97.

4. Uyehara,C.M. and McKay,D.J. (2019) Direct and widespread role for the nuclear receptor EcR in mediating the response to ecdysone in *Drosophila*. *Proc. Natl. Acad. Sci. U.S.A.*, **116**, 9893–9902.

5. Mazina,M.Yu., Kovalenko,E.V., Derevyanko,P.K., Nikolenko,J.V., Krasnov,A.N. and Vorobyeva,N.E. (2018) One signal stimulates different transcriptional activation mechanisms. *Biochimica et Biophysica Acta (BBA) - Gene Regulatory Mechanisms*, **1861**, 178–189.

6. Mazina,M.Y., Kovalenko,E.V. and Vorobyeva,N.E. (2021) The negative elongation factor NELF promotes induced transcriptional response of Drosophila ecdysone-dependent genes. *Scientific Reports*, **11**, 172.

7. Vorobyeva,N.E., Mazina,M.U., Golovnin,A.K., Kopytova,D.V., Gurskiy,D.Y., Nabirochkina,E.N., Georgieva,S.G., Georgiev,P.G. and Krasnov,A.N. (2013) Insulator protein Su(Hw) recruits SAGA and Brahma complexes and constitutes part of Origin Recognition Complex-binding sites in the Drosophila genome. *Nucleic Acids Res.*, **41**, 5717–5730.

8. Simon,J.M., Giresi,P.G., Davis,I.J. and Lieb,J.D. (2012) Using formaldehyde-assisted isolation of regulatory elements (FAIRE) to isolate active regulatory DNA. *Nature Protocols*, **7**, 256–267.

9. Ramírez,F., Ryan,D.P., Grüning,B., Bhardwaj,V., Kilpert,F., Richter,A.S., Heyne,S., Dündar,F. and Manke,T. (2016) deepTools2: a next generation web server for deep-sequencing data analysis. *Nucleic Acids Res.*, **44**, W160-165.

10. Feng,J., Liu,T., Qin,B., Zhang,Y. and Liu,X.S. (2012) Identifying ChIP-seq enrichment using MACS. *Nat Protoc*, **7**, 1728–1740.

11. Identifying ChIP-seq enrichment using MACS | Nature Protocols (2020).

12. Ross-Innes,C.S., Stark,R., Teschendorff,A.E., Holmes,K.A., Ali,H.R., Dunning,M.J., Brown,G.D., Gojis,O., Ellis,I.O., Green,A.R., *et al.* (2012) Differential oestrogen receptor binding is associated with clinical outcome in breast cancer. *Nature*, **481**, 389–393.

13. Afgan,E., Baker,D., Batut,B., van den Beek,M., Bouvier,D., Čech,M., Chilton,J., Clements,D., Coraor,N., Grüning,B.A., *et al.* (2018) The Galaxy platform for accessible, reproducible and collaborative biomedical analyses: 2018 update. *Nucleic Acids Res*, **46**, W537–W544.

14. Adato,O., Sloutskin,A., Komemi,H., Brabb,I., Duttke,S., Bucher,P., Unger,R. and Juven-Gershon,T. (2024) ElemeNT 2023: an enhanced tool for detection and curation of core promoter elements. *Bioinformatics*, **40**, btae110.

15. Dreos,R., Ambrosini,G., Groux,R., Cavin Périer,R. and Bucher,P. (2017) The eukaryotic promoter database in its 30th year: focus on non-vertebrate organisms. *Nucleic Acids Res*, **45**, D51–D55.

16. Mazina,M.Y., Nikolenko,J.V., Fursova,N.A., Nedil’ko,P.N., Krasnov,A.N. and Vorobyeva,N.E. (2015) Early-late genes of the ecdysone cascade as models for transcriptional studies. *Cell Cycle*, **14**, 3593–3601.
